# Phylogeography and genetic diversity of *Pieris rapae* across continents

**DOI:** 10.1371/journal.pone.0354257

**Published:** 2026-07-24

**Authors:** Md. Ahashan Habib Siam, D. Dey, J. K. Owaresat, Yousha Shamim Khan, Efrin Anowar, J. F. Maya, AMAM Zonaed Siddiki

**Affiliations:** 1 Department of Zoology, University of Chittagong, Chattogram, Bangladesh; 2 Department of Computer Science and Engineering, University of Chittagong, Chattogram, Bangladesh; 3 Department of Pathology and Parasitology, Faculty of Veterinary Medicine, Chattogram Veterinary and Animal Sciences University, Chattogram, Bangladesh; Sunrise University, INDIA

## Abstract

The small cabbage white butterfly (*Pieris rapae*) is a globally distributed agricultural pest that has successfully colonized most continents through natural and human-mediated dispersal. This study investigates the global genetic diversity and phylogeographic structure of *Pieris rapae* using mitochondrial cytochrome c oxidase subunit I (COI) sequences. A total of 1,133 sequences from five continents (Africa, Asia, Europe, North America, and Oceania) were analyzed to assess haplotype diversity, nucleotide diversity, population structure, and evolutionary relationships. Across the dataset, 117 haplotypes were identified, with overall high haplotype diversity (Hd = 0.8635) and moderate nucleotide diversity (π = 0.00610). Europe exhibited the highest genetic diversity, while North America showed low haplotype diversity but comparatively high nucleotide divergence, suggesting multiple introduction events. Minimum spanning network analysis revealed a star-like pattern with several widely shared central haplotypes, indicating recent expansion and extensive dispersal. Phylogenetic analysis identified two major mitochondrial clades, with North American haplotypes distributed across both clades. AMOVA results showed that most genetic variation occurred within populations (71.62%), although significant differentiation existed among continents (FST = 0.28385, p < 0.05). Pairwise FST and gene flow estimates indicated restricted intercontinental gene exchange, while the Mantel test showed no significant isolation by distance. Overall, the results suggest a complex invasion history shaped by historical introductions, demographic expansion, and human-mediated dispersal.

## 1. Introduction

Invasive species are a major component of global biodiversity change and pose significant threats to agriculture, ecosystems, and economies [1]. *Pieris rapae*, commonly known as the small cabbage white butterfly, is a highly abundant, migratory pest of cabbage that is among the most globally widespread and destructive butterfly species, causing severe damage worldwide [2–4]. It is considered among the most prevalent butterflies in temperate regions worldwide, including Europe, North America, North and East Asia, Australia, and New Zealand [5–7]. Recent research indicates that *Pieris rapae* originated in the Mediterranean and West Asia, reached East Asia around 1200 BP, and has spread globally, including to North America, Australia, and New Zealand, over the past 160 years [2]. Understanding their population genetic structure is essential for reconstructing invasion pathways, identifying source populations, and explaining the evolutionary processes that facilitate successful establishment in new environments.

Mitochondrial DNA (mtDNA) is a widely used molecular marker for assessing genetic diversity and population structure due to its maternal inheritance, lack of recombination, near-neutral evolution, relatively rapid mutation rate, and technical ease, making it particularly informative for studying phylogenetic relationships [8–11]. Among mitochondrial markers, the cytochrome c oxidase subunit I (COI) gene is widely employed for DNA barcoding, allowing accurate species identification and the evaluation of molecular diversity and phylogenetic relationships [12].

Despite the ecological importance of *Pieris rapae*, a comprehensive global-scale synthesis of its mitochondrial genetic diversity across multiple continents remains limited. Most previous studies have focused on regional populations or restricted geographic areas, leaving gaps in understanding its global invasion history and population connectivity. Integrating large-scale datasets across continents is therefore essential to clarify whether genetic structuring is primarily driven by historical colonization events, contemporary gene flow, or repeated anthropogenic introductions.

This study analyzed 1,133 mitochondrial COI sequences of *Pieris rapae* collected from Africa, Asia, Europe, North America, and Oceania to investigate global genetic diversity and population structure. The haplotype diversity, nucleotide diversity, and population differentiation were assessed using AMOVA and pairwise FST analyses, and reconstructed phylogeographic relationships using minimum spanning networks and maximum likelihood phylogenetic inference. In addition, this study tested for isolation by distance using Mantel analysis to evaluate the role of geographic separation in shaping genetic structure.

By integrating these approaches, this study aims to (i) quantify global patterns of genetic diversity in *Pieris rapae*, (ii) identify phylogeographic structure and major mitochondrial lineages, and (iii) infer historical and contemporary processes driving its global distribution. The results provide important insights into the invasion biology of *Pieris rapae* and contribute to a broader understanding of how invasive agricultural pests spread and diversify across continents under the combined influence of evolutionary and anthropogenic forces.

## 2. Materials and methods

### 2.1. Sample collection

#### 2.1.1. Sequence retrieval and sample selection.

Mitochondrial DNA (mtDNA), particularly the mitochondrial cytochrome c oxidase I (COI) gene widely used in DNA barcoding [13], is a powerful tool for species identification and evolutionary studies due to its maternal inheritance and relatively rapid mutation rate [14], which provide valuable insights into genetic diversity and phylogenetic structure [15, 16]. For this study, mitochondrial DNA (mtDNA) cytochrome c oxidase subunit I (COI) sequences of *Pieris rapae* were obtained from the NCBI GenBank database. A comprehensive search was conducted using the keywords “*Pieris rapae* COI,” “*Pieris rapae* CO1,” “*Pieris rapae* COX1,” and “*Pieris rapae* cytochrome c oxidase I,” and all relevant entries available up to February 2026 were examined.

Sequences were included only if they met the following criteria: (i) they were identified as *Pieris rapae* and annotated as mitochondrial cytochrome c oxidase subunit I (COI) in the NCBI GenBank database; (ii) they were at least 500 bp in length; (iii) they contained minimal ambiguous nucleotides (e.g., “N”) and showed no obvious sequencing errors; (iv) duplicate or redundant records were removed to avoid overrepresentation of identical sequence entries in downstream analyses; (v) sequences lacking geographic information, containing incomplete metadata, or having uncertain species identification were excluded; and (vi) only publicly available sequences submitted up to February 2026 were included. Using these criteria, a total of 1,133 mtDNA COI sequences (502–678 bp) were obtained from five continents: Africa, Asia, Europe, North America, and Oceania (NCBI accession number and geographic locations are provided in S1 Table). No sequences from the South America were found to analyze. This research did not involve any studies with human participants.

#### 2.1.2. Global sampling of COI sequences.

A total of 1,133 mitochondrial COI sequences of *Pieris rapae* were retrieved from the NCBI GenBank database to capture global genetic diversity. Among these, 46 sequences originated from Africa, 160 from Asia, 470 from Europe, 356 from North America, and 101 from Oceania. This sampling strategy ensured wide geographic coverage, enabling a comparative analysis of haplotype diversity across major continental regions ((NCBI accession numbers and corresponding geographic locations are listed in S1 Table).

### 2.2. Data analysis

#### 2.2.1. Genetic diversity and phylogeographic analyses.

Multiple sequence alignment of the 1,133 *Pieris rapae* mtDNA COI sequences was performed using MAFFT v7 [17]. The resulting alignment was manually inspected in BioEdit v7.7.1 [18], where poor-quality regions, ambiguous nucleotide positions, and uneven sequence termini were trimmed to produce a homologous alignment. Sequence quality and alignment accuracy were subsequently verified in MEGA v12 [19] before downstream analyses. Haplotype-based genetic diversity indices were estimated using DnaSP v6.12.05 [20]. A minimum spanning network (MSN) was subsequently constructed in PopART v1.7 [21] to illustrate relationships among haplotypes and their distribution patterns. Geographic distributions of haplotypes were mapped in R [22] using the maps package, incorporating spatial data from the CIA World Data Bank II [23]. Phylogeographic relationships were inferred using the Maximum Likelihood (ML) approach with 1,000 bootstrap replicates in RAxML-NG v1.0.3 [24]. The best-fit nucleotide substitution model (HKY + F + G) was selected using IQ-TREE v2.0.7 [25]. Final phylogenetic trees were visualized and annotated in iTOL v5 [26].

#### 2.2.2. Population structure analyses.

Population genetic structure across continental populations was assessed using Analysis of Molecular Variance (AMOVA) and pairwise FST estimates implemented in Arlequin v3.5.2.2 [27], with significance tested through 10,000 permutations. The association between genetic and geographic distances was further evaluated using a Mantel test [28] conducted in the vegan R package [29]. The statistical significance of the Mantel test was determined using 999 permutations. Genetic distances were computed under the Kimura two-parameter model using the *dist.dna* function from the ape R package, while geographic distances were derived from sampling coordinates based on the Haversine formula [30].

## 3. Results

### 3.1. Genetic diversity of *Pieris rapae* populations across different continents

A total of 1,133 COI sequences from five continents were examined to evaluate haplotype diversity and nucleotide variation in *Pieris rapae*. The sequence lengths varied among datasets, ranging from 502 to 678 base pairs. Analyses conducted separately for each continent showed substantial variation in both the number of haplotypes and their genetic diversity (Table 1). Europe exhibited the highest genetic diversity, with 75 haplotypes (Hd = 0.841) and the highest nucleotide diversity (π = 0.00487), indicating pronounced genetic differentiation. Asia also showed considerable diversity, with 33 haplotypes (Hd = 0.677) and moderate nucleotide diversity (π = 0.00313).

**Table 1:** Genetic diversity indices of *Pieris rapae* populations across different continents.

Sampling region	n	S	Eta	h	Hd	π	k
Africa	46	7	7	7	0.709	0.00230	1.155
Asia	160	29	30	33	0.677	0.00313	1.570
Europe	470	51	55	75	0.841	0.00487	2.447
North America	356	174	224	22	0.471	0.00633	3.180
Oceania	101	4	4	4	0.529	0.00252	1.267
Global	1133	185	251	117	0.8635	0.00610	3.063

(n = number of sequences, S = number of polymorphic sites, Eta = Total number of mutations, h = number of haplotypes, Hd = haplotype diversity, π = nucleotide diversity, and k = average number of nucleotide differences).

In contrast, Africa displayed moderate haplotype diversity (Hd = 0.709) but low nucleotide diversity (π = 0.00230), suggesting limited sequence divergence despite the presence of multiple haplotypes. Oceania showed relatively low diversity, with only 4 haplotypes (Hd = 0.529) and low nucleotide variation (π = 0.00252), reflecting a more homogeneous population structure. Interestingly, North America exhibited a contrasting pattern, with relatively low haplotype diversity (Hd = 0.471) despite having a high number of polymorphic sites (S = 174) and the highest nucleotide diversity (π = 0.00633). This indicates the presence of fewer but more divergent haplotypes within the population.

Following the integration of sequences from all five continents into a single dataset and subsequent multiple sequence alignment, a consensus alignment of 502 bp was generated. This length corresponds to the homologous region consistently retained across all samples for downstream analyses. A total of 185 polymorphic sites and 251 mutations were identified, resulting in 117 distinct haplotypes. Overall haplotype diversity was high (Hd = 0.8635), indicating extensive genetic variation at the global scale. Nucleotide diversity was also relatively elevated (π = 0.00610), with an average of 3.063 nucleotide differences among sequences.

Among the 117 haplotypes identified, Africa comprised 7 haplotypes (Hap_1 to Hap_7), Asia contained 33 haplotypes (Hap_4, Hap_8 to Hap_39), Europe showed the highest diversity with 75 haplotypes (Hap_1 to Hap_4, Hap_6, Hap_10, Hap_12 to Hap_13, Hap_15, Hap_28 to Hap_30, Hap_40 to Hap_102), North America included 22 haplotypes (Hap_1 to Hap_2, Hap_4, Hap_6, Hap_42, Hap_47, Hap_57, Hap_60, Hap_103 to Hap_116), and Oceania was represented by 4 haplotypes (Hap_1, Hap_4, Hap_6 and Hap_117). The variation in haplotype numbers among continents likely reflects differences in sampling effort and sequence representation across regions rather than true biological differences alone (Table 1, S2 Table).

**Fig 1:**
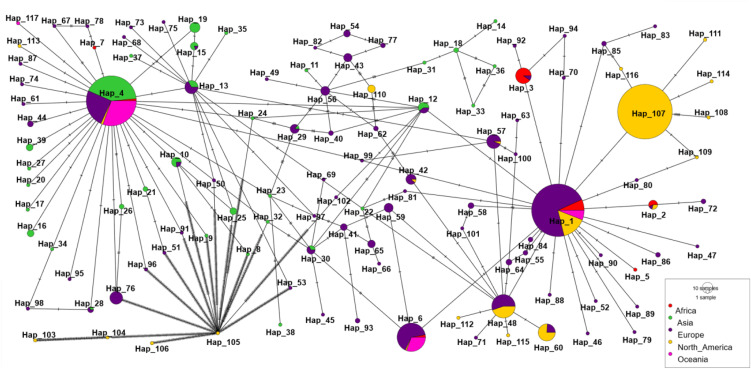
Minimum spanning network (MSN) of mtDNA COI haplotypes (Hap_1–Hap_117) in *Pieris rapae* populations across multiple continents. Each circle corresponds to a distinct haplotype, with its size proportional to haplotype frequency. Colors indicate continental origin, and black marks along the connecting branches represent the number of mutational steps separating haplotypes.

Given the large dataset (n = 1,133), haplotype relationships were inferred using a minimum spanning network (MSN) (Fig 1), which provides a computationally efficient and interpretable alternative to median-joining (MJ) networks for large-scale datasets. The minimum spanning network (MSN) of 117 haplotypes revealed a complex pattern of genetic relationships with limited geographic structuring across continents. Several high-frequency haplotypes occupied central positions, connecting numerous low-frequency haplotypes through relatively few mutational steps, consistent with widespread shared ancestry and ongoing gene flow. Notably, dominant haplotypes (e.g., Hap_1, Hap_4 and Hap_6) formed highly connected nodes comprising individuals from multiple continents, suggesting they represent ancestral or widely dispersed lineages. Peripheral haplotypes were often separated from common haplotypes by one or a few mutational steps, with some local hub-like patterns observed around high-frequency haplotypes, suggesting recent diversification. Europe contributed the largest proportion of individuals to the major haplotypes and shared the greatest number of haplotypes with other continents, highlighting its central role in global genetic connectivity, whereas Asia exhibited a broader distribution across numerous low-frequency haplotypes. Africa showed moderate representation, including both shared and continent-specific haplotypes, while fewer, more localized haplotypes represented North America and Oceania. The prevalence of mixed-origin nodes throughout the network further supports weak geographic structuring and high connectivity among global populations. Overall, these results highlight pronounced geographic variation in genetic diversity, with the global dataset reflecting high haplotype richness and moderate nucleotide divergence across populations.

### 3.2. Phylogeographic relationships among haplotypes of *Pieris rapae* populations across different continents

**Fig 2:**
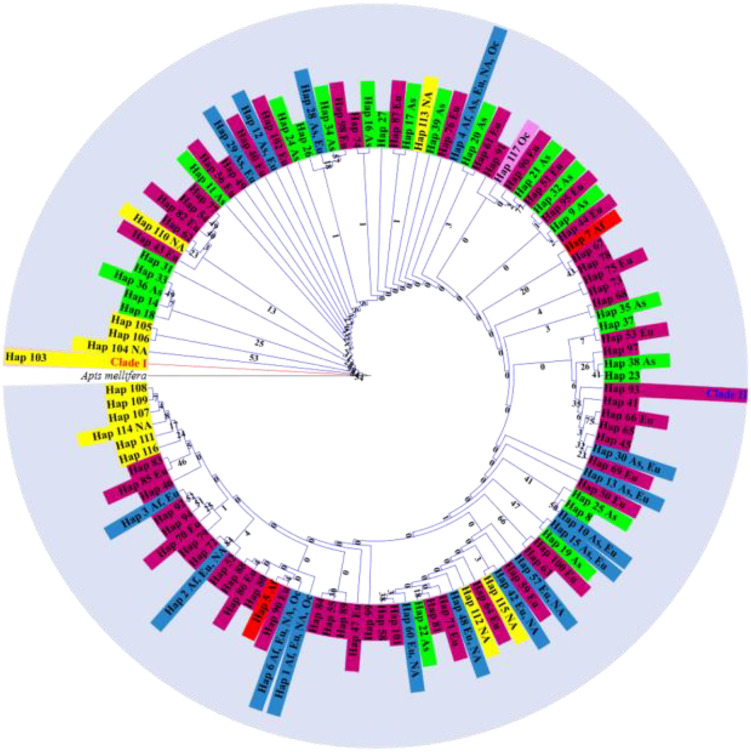
Maximum Likelihood phylogeographic tree constructed from mtDNA COI haplotypes of global *Pieris rapae* populations. Haplotypes are categorized based on their continental origin, with bootstrap support values indicated at the nodes. *Apis mellifera* was used as the outgroup. Continental labels are as follows: Africa = Af (red), Asia = As (green), Europe = Eu (purple), North America = NA (yellow), Oceania = Oc (pink), and Shared = Blue.

A Maximum Likelihood phylogenetic analysis identified two major mitochondrial haplotype clades among *Pieris rapae* mellifera samples collected from five continents (Fig 2). Interestingly, haplotypes from North American populations were divided into two distinct clades: Hap_107 grouped within clade I, while the remaining haplotypes clustered in clade II alongside haplotypes from other continents. The Minimum Spanning Network (MSN) (Fig 1) further supports this phylogeographic pattern.

### 3.3. Population differentiation and molecular variance analysis

**Fig 3:**
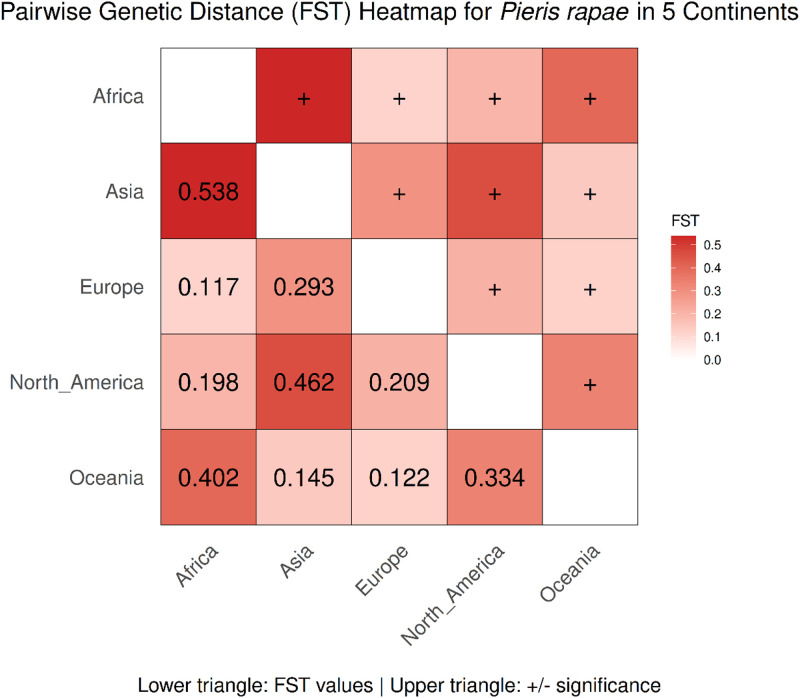
Pairwise genetic differentiation (FST) among continental populations of *Pieris rapae* based on mtDNA COI sequences, with FST values shown in the lower triangle and corresponding P-values in the upper triangle; significant differentiation (P ≤ 0.05) is indicated by “+”.

**Fig 4:**
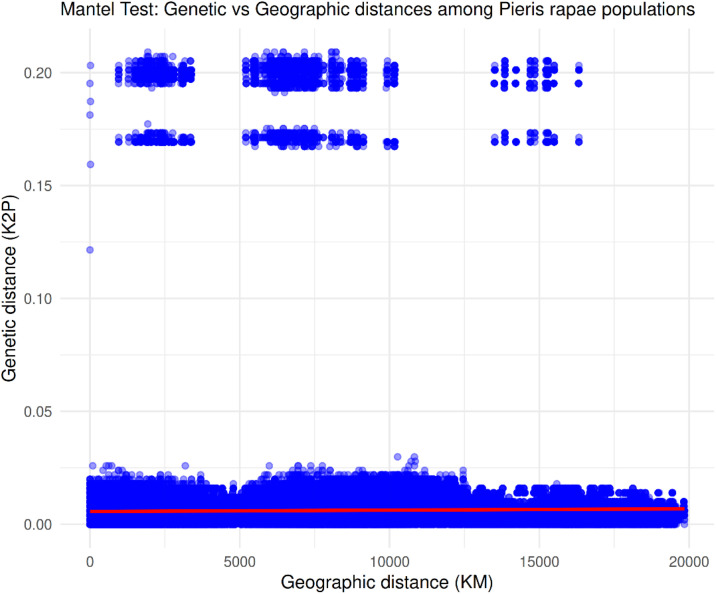
Relationship between genetic and geographic distances among 1,133 *Pieris rapae* sequences. Pairwise genetic distances were calculated using the Kimura 2-parameter model, and geographic distances were estimated using the Haversine formula. The slope of the red regression line indicates that geographic separation does not strongly predict genetic differentiation across global populations.

The population genetic structure across Africa, Asia, Europe, North America, and Oceania was investigated using Analysis of Molecular Variance (AMOVA), complemented by pairwise estimates of genetic differentiation (FST) to assess variation among populations. AMOVA revealed that most of the genetic variation in *Pieris rapae* populations was distributed within populations (71.62%), while a smaller but substantial proportion was attributed to variation among continental populations (28.38%) (P < 0.001). The overall fixation index (FST = 0.28385) indicates substantial genetic differentiation among continental populations of *Pieris rapae*, suggesting limited gene flow and clear population structuring (P < 0.05) (Table 2). All pairwise FST comparisons among population pairs were statistically significant, indicating consistent genetic differentiation across all populations. Among all pairwise comparisons, the highest genetic differentiation was observed between the African and Asian populations (FST = 0.538), while the lowest differentiation occurred between the African and European populations (FST = 0.117) (Fig 3). The estimated gene flow (Nm ≈ 0.63 migrants per generation) suggests restricted inter-population migration, indicating that gene exchange among continental populations is relatively low. The Mantel test revealed a very weak and non-significant correlation between genetic and geographic distances (r = 0.01797, p = 0.18) (Table 3), indicating no evidence of isolation by distance among populations of *Pieris rapae*. The corresponding scatterplot (Fig 4) shows that geographic separation does not significantly explain the observed genetic differentiation across continental populations.

**Table 2:** AMOVA results showing genetic variation among continental populations of *Pieris rapae* based on mtDNA COI sequences.

Source of variation	Degrees of freedom (df)	Sum of squares	Variance components	Percentage of variation	Fixation index (FST)	P-value	Gene flow (Nm)
Among populations	4	381.491	0.47520	28.38%	0.28385	0.00000	0.63
Within populations	1128	1352.401	1.19894	71.62%			
Total	1133	1733.892	1.67414	100%			

(Significant at P < 0.001).

**Table 3:** Mantel Test results for *Pieris rapae* among continental populations.

Statistic	Value	Significance (P-value)	Method	Permutation
Mantel statistic (r)	0.01797	0.18	pearson	999

(Significant at P < 0.05).

## 4. Discussion

The small cabbage white butterfly (*Pieris rapae*) is a major pest of cruciferous crops and, through human-mediated dispersal, has established populations on all continents except South America and Antarctica [2]. Assessing global genetic variation is a crucial component of modern biosecurity and invasive species management. By analyzing the genetic makeup of invasive populations, researchers can trace their origins, understand how they spread, and predict their potential impacts, leading to more effective, targeted control strategies. Haplotype diversity (Hd) and nucleotide diversity (π) are commonly used measures of genetic variation within and among populations, capturing the combined effects of mutation, genetic drift, and gene flow [13,31]. Mitochondrial DNA (mtDNA) is widely used in species identification and population genetic studies due to its maternal inheritance, relatively rapid evolutionary rate, extensive reference datasets, and informative properties for investigating genetic diversity, phylogeography, and evolutionary relationships [32–34].

The present study reveals substantial global genetic variation in *Pieris rapae*, with pronounced differences in haplotype and nucleotide diversity among continents. Europe exhibited the highest levels of genetic diversity (Hd = 0.841, π = 0.00487), suggesting that it represents an important center of genetic variation and may have contributed to the global distribution of some maternal lineages. This pattern is consistent with expectations for native [2] or long-established populations, where prolonged evolutionary history, large effective population sizes, and continuous gene flow contribute to the accumulation of genetic diversity. These findings are in agreement with previous studies that identified the Mediterranean–West Asian region as the native range of *Pieris rapae* and proposed Europe as an important contributor to its subsequent global colonization through natural and human-mediated dispersal [2]. In contrast, Asia displayed moderate haplotype diversity (Hd = 0.677, π = 0.00313) distributed across numerous low-frequency haplotypes, suggesting ongoing diversification and possible regional sub-structuring. Populations from Africa and Oceania showed comparatively lower genetic diversity, which may reflect a combination of limited sampling and historical founder effects. Interestingly, North America exhibited a contrasting genetic pattern, with low haplotype diversity (Hd = 0.471) but the highest nucleotide diversity (π = 0.00633). This suggests the presence of fewer but highly divergent haplotypes, potentially resulting from multiple introduction events from genetically distinct source populations followed by limited admixture. Repeated introductions of invasive species do not necessarily result in increased genetic diversity or haplotype richness, as founder effects and genetic drift may constrain variation [35].

An important consideration when interpreting these continental patterns is the unequal sampling effort among regions. Europe and North America were represented by substantially larger numbers of COI sequences than Africa and Oceania, increasing the likelihood of detecting both common and rare haplotypes in these regions. Consequently, differences in haplotype richness may partly reflect variation in sampling intensity rather than biological differences alone. Although haplotype diversity (Hd) and nucleotide diversity (π) are generally less sensitive to sample size than the absolute number of haplotypes, comparisons among continents should nevertheless be interpreted with caution. Future studies incorporating more balanced geographic sampling, particularly from underrepresented regions, would provide a more comprehensive understanding of the global genetic diversity and phylogeographic structure of *Pieris rapae*.

At the global scale, the high haplotype diversity and moderate nucleotide diversity indicate a complex evolutionary history shaped by mutation, dispersal, and demographic processes. The minimum spanning network further supports this interpretation, revealing a star-like structure with several high-frequency, centrally located haplotypes connected to numerous low-frequency derivatives. The widespread distribution of dominant haplotypes (e.g., Hap_1, Hap_4, and Hap_6) across multiple continents suggests that these lineages may represent ancestral or widely dispersed maternal lineages that have spread globally, likely facilitated by human-mediated dispersal [2]. Phylogenetic analysis identified two major mitochondrial clades, further supporting the presence of genetic structuring within the species. The division of North American haplotypes into two clades is consistent with multiple introduction events or contributions from divergent maternal source populations. However, these interpretations should be considered tentative because mitochondrial COI represents only the maternal evolutionary history of the species. The concordance between phylogenetic and network analyses strengthens the inference of complex colonization pathways and historical dispersal patterns.

Despite the apparent global connectivity observed in the haplotype network, AMOVA results indicate that a substantial proportion of genetic variation (28.38%) is partitioned among continental populations, with a significant fixation index (FST = 0.28385). This suggests that, while gene flow has occurred historically, contemporary populations are genetically differentiated. The consistently significant pairwise FST values further support restricted gene exchange among continents. The relatively low estimated gene flow (Nm ≈ 0.63) reinforces this conclusion, indicating limited ongoing migration between populations.

Notably, the absence of a significant isolation-by-distance pattern suggests that geographic distance alone does not explain genetic differentiation in *Pieris rapae*. Instead, the observed structure is more likely driven by historical colonization events, human-mediated dispersal, and localized demographic processes rather than gradual spatial separation. This pattern is characteristic of invasive species with complex introduction histories, where long-distance dispersal overrides the effects of geographic distance.

Overall, the findings of this study highlight the combined influence of historical introductions, demographic processes, and human activity in shaping the global mitochondrial genetic structure of *Pieris rapae*. The high genetic diversity observed in Europe, together with the widespread occurrence of shared haplotypes and relatively weak phylogeographic structure, is consistent with Europe representing an important center of genetic diversity and a potential contributor to the distribution of maternal lineages across continents. Nevertheless, because this study is based exclusively on mitochondrial COI sequences, the inferred invasion history and source populations should be interpreted cautiously. Future studies integrating nuclear genomic markers and more balanced geographic sampling will provide a more comprehensive understanding of the species’ dispersal history, admixture, and population structure.

## 5. Conclusion

This study provides a comprehensive overview of the global genetic structure of *Pieris rapae* based on mitochondrial COI data. The species exhibits high global haplotype diversity with moderate nucleotide variation, reflecting a complex evolutionary and invasion history. Europe appears to represent a major reservoir of genetic diversity, while North American populations show signatures of multiple introductions and divergent source lineages. Despite widespread sharing of dominant haplotypes across continents, significant genetic differentiation among populations indicates restricted gene flow following establishment. The absence of isolation by distance further suggests that historical colonization events and human-mediated dispersal, rather than geographic proximity, have primarily shaped current genetic patterns. Overall, these findings highlight the dynamic population history of *P. rapae* and provide valuable insights for understanding its global spread and for informing future pest management strategies.

## Supporting information

S1 Table NCBI accession information for 1133 mitochondrial COI sequences of *Pieris rapae* from the five continents. (ODT)

S2 Table Frequency and distribution of mtDNA COI haplotypes of *Pieris rapae* populations across five continents. (DOCX)
