# Supplementary material for: Phylogeography and genetic diversity of Pieris rapae across continents
Source: PLoS One. 2026 Jul 24;21(7):e0354257. doi: 10.1371/journal.pone.0354257 (PMC13399322; doi:10.1371/journal.pone.0354257)
Supplement: S2 Table — (DOCX) [file pone.0354257.s002.docx]

**S2 Table:** Frequency and distribution of mtDNA COI haplotypes of *Pieris rapae* populations across five continents.

Input Data File: C:\...\Combined_final.nex

Number of sequences: 1133 Number of sequences used: 1133

Selected region: 1-502 Number of sites: 502

Total number of sites (excluding sites with gaps / missing data): 502

Number of polymorphic (segregating) sites, S: 185

Total number of mutations, Eta: 251

Number of Haplotypes, h: 117

Haplotype (gene) diversity, Hd: 0.8635

Variance of Haplotype diversity: 0.0000308

Standard Deviation of Haplotype diversity: 0.0055

Nucleotide diversity, Pi: 0.00610

Sampling variance of Pi: n.d.

Standard deviation of Pi: n.d.

Theta (per site) from Eta: 0.06571

Theta (per site) from S, Theta-W: 0.04843

Variance of theta (no recombination): 0.0000771

Standard deviation of theta (no recombination): 0.00878

Variance of theta (free recombination): 0.0000127

Standard deviation of theta (free recombination): 0.00356

Finite Sites Model

Theta (per site) from Pi: 0.00615

Theta (per site) from S: 0.06001

Theta (per site) from Eta: 0.07300

Average number of nucleotide differences, k: 3.063

Stochastic variance of k (no recombination), Vst(k): 2.541

Sampling variance of k (no recombination), Vs(k): 0.004

Total variance of k (no recombination), V(k): 2.545

Stochastic variance of k (free recombination), Vst(k): 1.021

Sampling variance of k (free recombination), Vs(k): 0.002

Total variance of k (free recombination), V(k): 1.023

Theta (per sequence) from S, Theta-W: 24.312

Variance of theta (no recombination): 19.426

Variance of theta (free recombination): 3.195

--------

Input Data File: C:\...\Combined_final_fixed.nex

Number of sequences: 1133 Number of sequences used: 1133

Selected region: 1-502 Number of sites: 502

Total number of sites (excluding sites with gaps / missing data): 502

Sites with alignment gaps: considered

Number of variable sites: 185

=========== Haplotype Distribution ===========

Number of haplotypes, h: 117

Haplotype diversity, Hd: 0.8635

Hap_1: 236 [1-2 5 8 11-12 16 18 26 29 31 35 37 41 45-46 209 211-212 214 216-217 221 225 228 239 241 243 247 250 252 254-255 258 262-263 265-266 272 277-278 280-281 283-285 290 292-294 296 298-299 302 307 310 313 317-321 325-326 329 331-332 334 336 347 354 359 363-364 368 373-376 378-379 382-384 386 390 393 397 401-403 405 408-411 416 419 424 426 429 434 436 439 442 445 447-450 453 456 459 461-463 467-470 473 475 480-483 486 489 492 496 498 500 506 508 510 515 524-525 527 532-533 535 537-540 545 547-548 550-551 556 558 561-563 565-567 572 574-575 581-582 586 594-596 601 608 611 613-615 617 623 627 633-634 638 646 650 653-654 657 663 689 691 711 715 725-726 730 735 739 741 770 780-782 784-787 803 805 866 892 900-901 921 926 929 931 938 944 970 972 981 993 1000 1010 1033 1044-1045 1052 1055-1056 1070 1085 1087-1088 1098 1105 1108 1112]

Hap_2: 7 [3 7 14 19 226 910 913]

Hap_3: 21 [4 6 10 15 20-24 27-28 30 32-34 38 42-44 576 624]

Hap_4: 214 [9 17 40 47-50 54-56 58-60 62 65 67-71 75 80-82 84-86 88 91-95 97 99-102 106-110 115-116 118 120 128-129 131-132 136-140 143 148-151 156 158-160 165 167-170 174-176 178 181-185 187 189-192 195-198 200-202 204-205 210 213 218 223 229 236 257 264 269 305-306 330 337 356-357 381 385 387 394 413 417 430 435 437-438 451 454-455 471-472 479 487 493 509 528 530 542-544 546 549 570 573 590-591 603 612 645 652 659 666 672 675 722 748 936 1035 1037 1040-1041 1047 1049-1051 1053-1054 1057-1064 1067-1068 1074-1075 1077-1081 1084 1086 1090-1095 1101-1104 1106-1107 1109-1111 1113-1133]

Hap_5: 1 [13]

Hap_6: 69 [25 36 244 251 304 311 323-324 327 366 421 432 444 446 497 513 523 526 553 557 569 578-580 583 587 589 593 598 602 604 610 620-622 625 628 630-632 636-637 639-643 829 1034 1036 1038-1039 1042-1043 1046 1048 1065-1066 1071-1073 1076 1082-1083 1089 1096-1097 1099-1100]

Hap_7: 1 [39]

Hap_8: 1 [51]

Hap_9: 1 [52]

Hap_10: 8 [53 57 63 186 188 203 344 606]

Hap_11: 1 [61]

Hap_12: 10 [64 146 157 162 164 172 338 342 609 618]

Hap_13: 15 [66 73 123 141 163 220 237 256 271 303 348 466 511 661 670]

Hap_14: 1 [72]

Hap_15: 5 [74 112 153 177 349]

Hap_16: 4 [76 104-105 152]

Hap_17: 1 [77]

Hap_18: 2 [78 125]

Hap_19: 9 [79 113-114 122 130 147 154 161 166]

Hap_20: 1 [83]

Hap_21: 2 [87 96]

Hap_22: 1 [89]

Hap_23: 1 [90]

Hap_24: 1 [98]

Hap_25: 4 [103 142 144 155]

Hap_26: 2 [111 124]

Hap_27: 1 [117]

Hap_28: 3 [119 301 312]

Hap_29: 7 [121 207 260 335 495 501-502]

Hap_30: 6 [126-127 224 341 400 418]

Hap_31: 1 [133]

Hap_32: 1 [134]

Hap_33: 1 [135]

Hap_34: 1 [145]

Hap_35: 1 [171]

Hap_36: 1 [173]

Hap_37: 1 [179]

Hap_38: 1 [180]

Hap_39: 4 [193-194 199 206]

Hap_40: 2 [208 355]

Hap_41: 4 [215 412 414 541]

Hap_42: 9 [219 249 270 431 647 649 658 660 865]

Hap_43: 6 [222 282 286 395 514 629]

Hap_44: 3 [227 289 554]

Hap_45: 1 [230]

Hap_46: 1 [231]

Hap_47: 1 [232]

Hap_48: 47 [233 240 245 253 261 291 295 322 328 369 389 422 428 441 477 484 499 505 560 584 588 607 619 656 665 668 698 705 714 719 724 729 812 930 932 934-935 946 958 966-968 973 975 977 980 1018]

Hap_49: 1 [234]

Hap_50: 1 [235]

Hap_51: 1 [238]

Hap_52: 1 [242]

Hap_53: 1 [246]

Hap_54: 4 [248 259 333 440]

Hap_55: 2 [267 273]

Hap_56: 7 [268 380 406 443 478 564 599]

Hap_57: 17 [274-276 396 452 476 485 516 519 531 534 536 555 651 667 669 907]

Hap_58: 3 [279 488 655]

Hap_59: 7 [287 314-316 433 577 600]

Hap_60: 25 [288 297 377 626 671 676 684 697 699 707-708 731-732 802 807 873 898-899 903-905 916 939 979 1030]

Hap_61: 1 [300]

Hap_62: 2 [308 559]

Hap_63: 1 [309]

Hap_64: 3 [339 361 503]

Hap_65: 5 [340 345-346 350 358]

Hap_66: 1 [343]

Hap_67: 1 [351]

Hap_68: 1 [352]

Hap_69: 1 [353]

Hap_70: 1 [360]

Hap_71: 1 [362]

Hap_72: 2 [365 398]

Hap_73: 1 [367]

Hap_74: 1 [370]

Hap_75: 1 [371]

Hap_76: 14 [372 388 404 415 420 423 425 460 465 504 507 512 518 520]

Hap_77: 2 [391 490]

Hap_78: 1 [392]

Hap_79: 1 [399]

Hap_80: 1 [407]

Hap_81: 1 [427]

Hap_82: 1 [457]

Hap_83: 1 [458]

Hap_84: 2 [464 529]

Hap_85: 2 [474 552]

Hap_86: 2 [491 517]

Hap_87: 1 [494]

Hap_88: 1 [521]

Hap_89: 1 [522]

Hap_90: 1 [568]

Hap_91: 1 [571]

Hap_92: 1 [585]

Hap_93: 2 [592 597]

Hap_94: 1 [605]

Hap_95: 1 [616]

Hap_96: 1 [635]

Hap_97: 1 [644]

Hap_98: 1 [648]

Hap_99: 1 [662]

Hap_100: 1 [664]

Hap_101: 1 [673]

Hap_102: 1 [674]

Hap_103: 1 [677]

Hap_104: 1 [678]

Hap_105: 1 [679]

Hap_106: 1 [680]

Hap_107: 255 [681-682 685 688 690 692-696 700-704 706 709-710 712-713 716-718 720-721 723 727-728 733-734 736-738 740 742-747 749-769 771-779 783 788-801 804 806 808 810-811 815-828 830-858 860-864 868-872 874-878 880-891 893-894 896-897 902 906 908-909 911-912 914-915 918-920 922-925 927-928 933 937 940-943 945 947-957 959-965 969 971 974 976 978 982-992 994-999 1001-1009 1011-1017 1019-1020 1022-1029 1031]

Hap_108: 1 [683]

Hap_109: 1 [686]

Hap_110: 5 [687 859 879 917 1021]

Hap_111: 1 [809]

Hap_112: 1 [813]

Hap_113: 1 [814]

Hap_114: 1 [867]

Hap_115: 1 [895]

Hap_116: 1 [1032]

Hap_117: 1 [1069]

Hap_1: 236 [MN182318.1_Africa_1 MN182311.1_Africa_2 MN182314.1_Africa_5 MN182309.1_Africa_8 MH418735.1_Africa_11 MH418996.1_Africa_12 MH419498.1_Africa_16 MH419177.1_Africa_18 MN182302.1_Africa_26 MN182315.1_Africa_29 MN182317.1_Africa_31 MH419260.1_Africa_35 MH419186.1_Africa_38 MH419253.1_Africa_42 MH419084.1_Africa_46 MH419137.1_Africa_47 MN145333.1_Europe_3 MN144390.1_Europe_5 MN144242.1_Europe_6 MN142981.1_Europe_8 MN139621.1_Europe_10 MN139482.1_Europe_11 KC462816.1_Europe_15 OR891199.1_Europe_19 OR891196.1_Europe_22 OR891184.1_Europe_34 OR891182.1_Europe_36 OR891180.1_Europe_38 MN182182.1_Europe_42 MN182179.1_Europe_45 MN182177.1_Europe_47 MN182175.1_Europe_49 MN182174.1_Europe_50 MN182171.1_Europe_53 MN182167.1_Europe_57 MN181870.1_Europe_58 MN181868.1_Europe_60 MN181867.1_Europe_61 MN181861.1_Europe_67 MN181856.1_Europe_72 MN181855.1_Europe_73 MN181853.1_Europe_75 MN181852.1_Europe_76 MN181850.1_Europe_78 MN181849.1_Europe_79 MN181848.1_Europe_80 MN144711.1_Europe_85 MN143517.1_Europe_87 MN143210.1_Europe_88 MN142999.1_Europe_89 MN142773.1_Europe_91 MN142492.1_Europe_93 MN142439.1_Europe_94 MN141970.1_Europe_97 MN141075.1_Europe_102 MN139623.1_Europe_105 MN139131.1_Europe_108 MN138806.1_Europe_112 MN138788.1_Europe_113 MN138553.1_Europe_114 MN182319.1_Europe_115 MN182266.1_Europe_116 MN182239.1_Europe_120 MN182212.1_Europe_121 MH420062.1_Europe_124 MH419525.1_Europe_126 MH419453.1_Europe_127 MH419326.1_Europe_129 KX041897.1_Europe_131 MN182052.1_Europe_150 MH420231.1_Europe_157 MH419172.1_Europe_162 MW503297.1_Europe_176 MW503094.1_Europe_177 MW500273.1_Europe_181 MN182254.1_Europe_186 MN182253.1_Europe_187 MN182251.1_Europe_188 MN182250.1_Europe_189 MN182248.1_Europe_191 MN182073.1_Europe_192 MW502323.1_Europe_195 MW500136.1_Europe_196 MW499669.1_Europe_197 MW499058.1_Europe_199 MN144969.1_Europe_203 MN144484.1_Europe_206 MN143992.1_Europe_210 MN143701.1_Europe_214 MN143639.1_Europe_215 MN143541.1_Europe_216 MN143334.1_Europe_218 MN142959.1_Europe_221 MN142949.1_Europe_222 MN142827.1_Europe_223 MN142677.1_Europe_224 MN141409.1_Europe_230 MN140348.1_Europe_234 MN139866.1_Europe_239 MN139727.1_Europe_241 MN139436.1_Europe_244 MN138870.1_Europe_249 MN138753.1_Europe_251 MN138554.1_Europe_254 MN138503.1_Europe_257 MN182285.1_Europe_260 MN182283.1_Europe_262 MN182284.1_Europe_263 MN182264.1_Europe_264 MN182245.1_Europe_265 MN182219.1_Europe_268 MH420352.1_Europe_271 MH420286.1_Europe_274 MH420274.1_Europe_276 MH420210.1_Europe_277 MH420205.1_Europe_278 MH420174.1_Europe_283 MH420095.1_Europe_284 MH420009.1_Europe_285 MH419962.1_Europe_286 MH419926.1_Europe_289 MH419865.1_Europe_291 MH419742.1_Europe_296 MH419727.1_Europe_297 MH419719.1_Europe_298 MH419654.1_Europe_299 MH419574.1_Europe_302 MH419529.1_Europe_305 MH419491.1_Europe_308 MH419199.1_Europe_312 MH419073.1_Europe_314 MH419040.1_Europe_316 MH418936.1_Europe_322 MH418908.1_Europe_324 MH418889.1_Europe_326 MH418833.1_Europe_331 MH418576.1_Europe_341 MH418624.1_Europe_342 MH418520.1_Europe_344 MN182216.1_Europe_358 MN182214.1_Europe_359 MH419828.1_Europe_361 MH419630.1_Europe_363 MH419690.1_Europe_364 MH419360.1_Europe_365 MH419099.1_Europe_366 MN182229.1_Europe_371 MN182227.1_Europe_373 MN182226.1_Europe_374 MN182224.1_Europe_376 MN182223.1_Europe_377 MN182213.1_Europe_382 MN182204.1_Europe_384 MN182200.1_Europe_387 MN182201.1_Europe_388 MN182202.1_Europe_389 HQ004962.1_Europe_391 HQ004960.1_Europe_392 HQ004959.1_Europe_393 HQ004954.1_Europe_398 MW502742.1_Europe_400 MW502012.1_Europe_401 MN141439.1_Europe_407 MN141230.1_Europe_408 MN139182.1_Europe_412 MN182235.1_Europe_420 MN182233.1_Europe_421 MN182215.1_Europe_422 MN182189.1_Europe_427 MN181889.1_Europe_434 GU669670.1_Europe_437 GU676736.1_Europe_439 GU676848.1_Europe_440 GU669615.1_Europe_441 GU669620.1_Europe_443 GU676400.1_Europe_449 GU675995.1_Europe_453 GU675844.1_Europe_459 MN138718.1_Europe_460 KP870897.1_Europe_464 MW501659.1_Europe_472 MW499555.1_Europe_476 MN141416.1_Europe_479 MN182295.1_Europe_480 MN182279.1_Europe_483 MN182273.1_Europe_489 MN181938.1_North_America_16 MN181762.1_North_America_18 KM545294.1_North_America_38 JF841240.1_North_America_48 KT127776.1_North_America_73 KX281214.1_North_America_75 HQ583581.1_North_America_79 MN182328.1_North_America_84 MN182323.1_North_America_88 MN182321.1_North_America_90 MN182140.1_North_America_119 MN182130.1_North_America_129 MN182129.1_North_America_130 MN182128.1_North_America_131 MN182126.1_North_America_133 MN182125.1_North_America_134 MN182124.1_North_America_135 MN182123.1_North_America_136 MN182011.1_North_America_152 MN182008.1_North_America_154 MN181905.1_North_America_215 MN181778.1_North_America_241 MN181770.1_North_America_249 MN181769.1_North_America_250 MN181741.1_North_America_270 MN181740.1_North_America_275 MN181733.1_North_America_278 MN181731.1_North_America_280 MN181724.1_North_America_287 MN181718.1_North_America_293 MN181686.1_North_America_319 MN181688.1_North_America_321 MN181672.1_North_America_330 MN181665.1_North_America_342 MN181652.1_North_America_349 MN181642.1_North_America_359 JN276270.1_Oceania_1 MN181999.1_Oceania_13 MN181998.1_Oceania_14 MN181956.1_Oceania_21 MN181953.1_Oceania_24 MN181952.1_Oceania_25 MN181839.1_Oceania_39 MN181815.1_Oceania_54 MN181813.1_Oceania_56 MN181812.1_Oceania_57 MN181801.1_Oceania_67 MN181794.1_Oceania_74 KF396514.1_Oceania_78 MN181988.1_Oceania_82]

Hap_2: 7 [MN182312.1_Africa_3 MN182308.1_Africa_7 MH419632.1_Africa_14 MH418626.1_Africa_19 OR891198.1_Europe_20 MN181752.1_North_America_259 MN181747.1_North_America_262]

Hap_3: 21 [MN182313.1_Africa_4 MN182307.1_Africa_6 MH418536.1_Africa_10 MH419399.1_Africa_15 MN182296.1_Africa_20 MN182297.1_Africa_21 MN182298.1_Africa_22 MN182299.1_Africa_23 MN182300.1_Africa_24 MN182301.1_Africa_27 MN182304.1_Africa_28 MN182316.1_Africa_30 MN182310.1_Africa_32 MN182305.1_Africa_33 MN182306.1_Africa_34 MH419959.1_Africa_39 MH419320.1_Africa_43 MH418702.1_Africa_44 MH418814.1_Africa_45 GU675871.1_Europe_402 GU676446.1_Europe_450]

Hap_4: 214 [MH420349.1_Africa_9 MH419165.1_Africa_17 MH419503.1_Africa_41 MN182095.1_Asia_1 MN182094.1_Asia_2 MN182093.1_Asia_3 MN182092.1_Asia_4 MN182088.1_Asia_8 MN182087.1_Asia_9 MN182086.1_Asia_10 MN182084.1_Asia_12 MN182083.1_Asia_13 MN182082.1_Asia_14 MN182080.1_Asia_16 MN182077.1_Asia_19 MN182074.1_Asia_21 MN182075.1_Asia_22 MN182069.1_Asia_23 MN182068.1_Asia_24 MN182067.1_Asia_25 MN181885.1_Asia_29 MN181970.1_Asia_34 MN181969.1_Asia_35 MN181968.1_Asia_36 MN181966.1_Asia_38 MN181965.1_Asia_39 GU696024.1_Asia_40 EU105304.1_Asia_42 EU105297.1_Asia_45 EU105298.1_Asia_46 EU105299.1_Asia_47 EU105300.1_Asia_48 EU105301.1_Asia_49 EU105295.1_Asia_51 EU105293.1_Asia_53 EU105292.1_Asia_54 EU105291.1_Asia_55 EU105290.1_Asia_56 EU105286.1_Asia_60 EU105285.1_Asia_61 EU105284.1_Asia_62 EU105283.1_Asia_63 EU105282.1_Asia_64 EU105281.1_Asia_69 EU105272.1_Asia_70 EU105274.1_Asia_72 EU105276.1_Asia_74 EU105264.1_Asia_82 EU105265.1_Asia_83 EU105261.1_Asia_85 EU105260.1_Asia_86 EU105252.1_Asia_90 EU105253.1_Asia_91 EU105254.1_Asia_92 EU105255.1_Asia_93 EU105256.1_Asia_94 EU105249.1_Asia_97 EU105244.1_Asia_102 EU105245.1_Asia_103 EU105246.1_Asia_104 EU105232.1_Asia_105 EU105237.1_Asia_110 EU105239.1_Asia_112 EU105240.1_Asia_113 EU105241.1_Asia_114 EU105226.1_Asia_119 EU105228.1_Asia_121 EU105229.1_Asia_122 EU105230.1_Asia_123 EU105231.1_Asia_124 EU105220.1_Asia_128 EU105221.1_Asia_129 EU105213.1_Asia_130 EU105215.1_Asia_132 JX242475.1_Asia_135 JX242474.1_Asia_136 MN182106.1_Asia_137 MN182105.1_Asia_138 MN182104.1_Asia_139 MN182102.1_Asia_141 MN182100.1_Asia_143 MN182099.1_Asia_144 MN182098.1_Asia_145 MN182097.1_Asia_146 MN182036.1_Asia_149 MN182035.1_Asia_150 MN182034.1_Asia_151 MN182032.1_Asia_152 MN182030.1_Asia_154 MN182029.1_Asia_155 MN182028.1_Asia_156 MN182026.1_Asia_158 MN182025.1_Asia_159 MN144447.1_Europe_4 MN144127.1_Europe_7 KM573655.1_Europe_12 OR891201.1_Europe_17 OR891195.1_Europe_23 OR891188.1_Europe_30 MN182172.1_Europe_52 MN181869.1_Europe_59 MN181864.1_Europe_64 MN141407.1_Europe_100 MN141067.1_Europe_101 MH419923.1_Europe_125 MN182064.1_Europe_140 MH419873.1_Europe_159 MH419592.1_Europe_160 MW502548.1_Europe_194 MW499237.1_Europe_198 MN145418.1_Europe_200 MN144128.1_Europe_207 MN142249.1_Europe_227 MN141163.1_Europe_231 MN139309.1_Europe_245 MN138792.1_Europe_250 MN138593.1_Europe_252 MN138559.1_Europe_253 MN182238.1_Europe_266 MH420397.1_Europe_269 MH420391.1_Europe_270 MH419953.1_Europe_287 MH419942.1_Europe_288 MH419770.1_Europe_295 MH419559.1_Europe_303 MH419374.1_Europe_309 MH418914.1_Europe_325 MH418515.1_Europe_345 MN182218.1_Europe_356 MW500203.1_Europe_368 MN182231.1_Europe_369 MN182230.1_Europe_370 MN182228.1_Europe_372 MN182225.1_Europe_375 HQ004956.1_Europe_396 HQ004955.1_Europe_399 MN182243.1_Europe_416 MN182237.1_Europe_417 MN182187.1_Europe_429 GU669669.1_Europe_438 MW502038.1_Europe_471 MN143974.1_Europe_478 MN182277.1_Europe_485 MN182268.1_Europe_492 MN182194.1_Europe_498 MN181900.1_Europe_501 KT140090.1_North_America_61 MN182162.1_North_America_97 MN181730.1_North_America_285 MN182043.1_Oceania_4 MN182039.1_Oceania_6 MN182016.1_Oceania_9 MN182014.1_Oceania_10 MN181993.1_Oceania_16 MN181991.1_Oceania_18 MN181990.1_Oceania_19 MN181989.1_Oceania_20 MN181955.1_Oceania_22 MN181954.1_Oceania_23 MN181951.1_Oceania_26 MN181950.1_Oceania_27 MN181949.1_Oceania_28 MN181948.1_Oceania_29 MN181947.1_Oceania_30 MN181875.1_Oceania_31 MN181874.1_Oceania_32 MN181845.1_Oceania_33 MN181844.1_Oceania_36 MN181843.1_Oceania_37 MN181835.1_Oceania_43 MN181834.1_Oceania_44 MN181823.1_Oceania_46 MN181822.1_Oceania_47 MN181821.1_Oceania_48 MN181820.1_Oceania_49 MN181819.1_Oceania_50 MN181816.1_Oceania_53 MN181814.1_Oceania_55 MN181809.1_Oceania_59 MN181808.1_Oceania_60 MN181807.1_Oceania_61 MN181806.1_Oceania_62 MN181805.1_Oceania_63 MN181804.1_Oceania_64 MN181798.1_Oceania_70 MN181797.1_Oceania_71 MN181796.1_Oceania_72 MN181795.1_Oceania_73 MN181793.1_Oceania_75 KF404991.1_Oceania_76 MN182050.1_Oceania_79 MN182049.1_Oceania_80 MN182048.1_Oceania_81 MN181987.1_Oceania_83 MN182019.1_Oceania_84 MN181964.1_Oceania_85 MN181957.1_Oceania_86 MN181958.1_Oceania_87 MN181960.1_Oceania_88 MN181959.1_Oceania_89 MN181961.1_Oceania_90 MN181962.1_Oceania_91 MN181963.1_Oceania_92 MN181846.1_Oceania_93 MN181832.1_Oceania_94 MN181831.1_Oceania_95 MN181830.1_Oceania_96 MN181829.1_Oceania_97 MN181828.1_Oceania_98 MN181827.1_Oceania_99 MN181826.1_Oceania_100 MN181825.1_Oceania_101 MN181824.1_Oceania_102 MN181810.1_Oceania_103]

Hap_5: 1 [MH419526.1_Africa_13]

Hap_6: 69 [MN182303.1_Africa_25 MH419423.1_Africa_36 MW501122.1_Europe_39 MN182178.1_Europe_46 MN141637.1_Europe_99 MN139564.1_Europe_106 MN182242.1_Europe_118 MN182240.1_Europe_119 MN182208.1_Europe_122 MW501547.1_Europe_179 MN140162.1_Europe_236 MN139282.1_Europe_247 MN182286.1_Europe_259 MN182282.1_Europe_261 MH419139.1_Europe_313 MH418854.1_Europe_329 MH418636.1_Europe_340 MH418575.1_Europe_343 MN182221.1_Europe_379 MN182205.1_Europe_383 HQ004957.1_Europe_395 MN144940.1_Europe_404 MN144664.1_Europe_405 MN143579.1_Europe_406 MN141198.1_Europe_409 MN138573.1_Europe_413 MN182244.1_Europe_415 MN182234.1_Europe_419 MN182206.1_Europe_424 MN182185.1_Europe_428 MN182186.1_Europe_430 MN181887.1_Europe_436 GU676719.1_Europe_446 GU676534.1_Europe_447 GU676461.1_Europe_448 GU676452.1_Europe_451 GU675996.1_Europe_454 GU676386.1_Europe_456 GU675686.1_Europe_457 GU675841.1_Europe_458 KP871138.1_Europe_462 KP870909.1_Europe_463 KP870788.1_Europe_465 KP870597.1_Europe_466 KP870579.1_Europe_467 KP870279.1_Europe_468 KP870276.1_Europe_469 MN181972.1_North_America_178 MN182044.1_Oceania_3 MN182042.1_Oceania_5 MN182038.1_Oceania_7 MN182020.1_Oceania_8 MN182001.1_Oceania_11 MN182000.1_Oceania_12 MN181997.1_Oceania_15 MN181992.1_Oceania_17 MN181841.1_Oceania_34 MN181842.1_Oceania_35 MN181838.1_Oceania_40 MN181837.1_Oceania_41 MN181836.1_Oceania_42 MN181833.1_Oceania_45 MN181818.1_Oceania_51 MN181817.1_Oceania_52 MN181811.1_Oceania_58 MN181803.1_Oceania_65 MN181802.1_Oceania_66 MN181800.1_Oceania_68 MN181799.1_Oceania_69]

Hap_7: 1 [MH420018.1_Africa_40]

Hap_8: 1 [MN182091.1_Asia_5]

Hap_9: 1 [MN182090.1_Asia_6]

Hap_10: 8 [MN182089.1_Asia_7 MN182085.1_Asia_11 MN182079.1_Asia_17 MN182103.1_Asia_140 MN182101.1_Asia_142 MN182027.1_Asia_157 MN182057.1_Europe_147 MN181891.1_Europe_432]

Hap_11: 1 [MN182081.1_Asia_15]

Hap_12: 10 [MN182078.1_Asia_18 EU105242.1_Asia_100 EU105238.1_Asia_111 EU105223.1_Asia_116 EU105225.1_Asia_118 EU105218.1_Asia_126 MN182063.1_Europe_141 MN182059.1_Europe_145 MN181888.1_Europe_435 GU676602.1_Europe_444]

Hap_13: 15 [MN182076.1_Asia_20 MN182065.1_Asia_27 EU105269.1_Asia_77 EU105247.1_Asia_95 EU105224.1_Asia_117 KC462817.1_Europe_14 OR891187.1_Europe_31 MN182173.1_Europe_51 MN181862.1_Europe_66 MN141703.1_Europe_98 MN182053.1_Europe_151 MH420169.1_Europe_282 MH418861.1_Europe_327 MN182275.1_Europe_487 MN182247.1_Europe_496]

Hap_14: 1 [MN182066.1_Asia_26]

Hap_15: 5 [MN181886.1_Asia_28 EU105278.1_Asia_66 EU105234.1_Asia_107 EU105214.1_Asia_131 MN182054.1_Europe_152]

Hap_16: 4 [MN181884.1_Asia_30 EU105288.1_Asia_58 EU105287.1_Asia_59 EU105233.1_Asia_106]

Hap_17: 1 [MN182072.1_Asia_31]

Hap_18: 2 [MN182071.1_Asia_32 EU105271.1_Asia_79]

Hap_19: 9 [MN182070.1_Asia_33 EU105279.1_Asia_67 EU105280.1_Asia_68 EU105268.1_Asia_76 EU105266.1_Asia_84 EU105243.1_Asia_101 EU105235.1_Asia_108 EU105222.1_Asia_115 EU105227.1_Asia_120]

Hap_20: 1 [MN181967.1_Asia_37]

Hap_21: 2 [EU105305.1_Asia_41 EU105296.1_Asia_50]

Hap_22: 1 [EU105303.1_Asia_43]

Hap_23: 1 [EU105302.1_Asia_44]

Hap_24: 1 [EU105294.1_Asia_52]

Hap_25: 4 [EU105289.1_Asia_57 EU105248.1_Asia_96 EU105250.1_Asia_98 EU105236.1_Asia_109]

Hap_26: 2 [EU105277.1_Asia_65 EU105270.1_Asia_78]

Hap_27: 1 [EU105273.1_Asia_71]

Hap_28: 3 [EU105275.1_Asia_73 MN142102.1_Europe_96 MN139542.1_Europe_107]

Hap_29: 7 [EU105267.1_Asia_75 MW502246.1_Europe_1 MN182169.1_Europe_55 MH418874.1_Europe_130 MH419298.1_Europe_311 MH419031.1_Europe_317 MH419016.1_Europe_318]

Hap_30: 6 [EU105262.1_Asia_80 EU105263.1_Asia_81 OR891200.1_Europe_18 MN182060.1_Europe_144 MN143758.1_Europe_213 MN140902.1_Europe_232]

Hap_31: 1 [EU105259.1_Asia_87]

Hap_32: 1 [EU105258.1_Asia_88]

Hap_33: 1 [EU105257.1_Asia_89]

Hap_34: 1 [EU105251.1_Asia_99]

Hap_35: 1 [EU105217.1_Asia_125]

Hap_36: 1 [EU105219.1_Asia_127]

Hap_37: 1 [EU105216.1_Asia_133]

Hap_38: 1 [JX445952.1_Asia_134]

Hap_39: 4 [MN182096.1_Asia_147 MN182037.1_Asia_148 MN182031.1_Asia_153 MN182024.1_Asia_160]

Hap_40: 2 [MW501076.1_Europe_2 MH419958.1_Europe_158]

Hap_41: 4 [MN139941.1_Europe_9 MN142252.1_Europe_226 MN142137.1_Europe_228 MW501927.1_Europe_367]

Hap_42: 9 [KC462818.1_Europe_13 MN182180.1_Europe_44 MN181863.1_Europe_65 MN139295.1_Europe_246 MW501412.1_Europe_473 MW500842.1_Europe_475 MN182278.1_Europe_484 MN182276.1_Europe_486 MN181904.1_North_America_214]

Hap_43: 6 [OR891202.1_Europe_16 MN181851.1_Europe_77 MN181847.1_Europe_81 MN144118.1_Europe_208 MH418842.1_Europe_330 GU675998.1_Europe_455]

Hap_44: 3 [OR891197.1_Europe_21 MW501754.1_Europe_84 MN182220.1_Europe_380]

Hap_45: 1 [OR891194.1_Europe_24]

Hap_46: 1 [OR891193.1_Europe_25]

Hap_47: 1 [OR891192.1_Europe_26]

Hap_48: 47 [OR891191.1_Europe_27 OR891183.1_Europe_35 MN182184.1_Europe_40 MN182176.1_Europe_48 MN182168.1_Europe_56 MN144671.1_Europe_86 MN142903.1_Europe_90 MN182265.1_Europe_117 MN182198.1_Europe_123 MW499843.1_Europe_182 MN145190.1_Europe_202 MN139968.1_Europe_237 MN139448.1_Europe_243 MN138523.1_Europe_256 MH419839.1_Europe_293 MH419639.1_Europe_300 MH419069.1_Europe_315 MH418965.1_Europe_321 MN182199.1_Europe_386 MN139651.1_Europe_410 MN138567.1_Europe_414 MN181890.1_Europe_433 GU676659.1_Europe_445 MN182280.1_Europe_482 MN182267.1_Europe_491 MN182270.1_Europe_494 MN181755.1_North_America_25 KM547147.1_North_America_32 HM415246.1_North_America_47 HM415245.1_North_America_55 KT135786.1_North_America_64 HQ583582.1_North_America_78 MN181996.1_North_America_161 MN181732.1_North_America_279 MN181726.1_North_America_281 MN181729.1_North_America_283 MN181728.1_North_America_284 MN181716.1_North_America_295 MN181702.1_North_America_307 MN181696.1_North_America_315 MN181694.1_North_America_316 MN181695.1_North_America_317 MN181683.1_North_America_322 MN181681.1_North_America_324 MN181677.1_North_America_326 MN181679.1_North_America_329 MN181631.1_North_America_367]

Hap_49: 1 [OR891190.1_Europe_28]

Hap_50: 1 [OR891189.1_Europe_29]

Hap_51: 1 [OR891185.1_Europe_33]

Hap_52: 1 [OR891181.1_Europe_37]

Hap_53: 1 [MN182183.1_Europe_41]

Hap_54: 4 [MN182181.1_Europe_43 MN182170.1_Europe_54 MH419376.1_Europe_128 MN138535.1_Europe_255]

Hap_55: 2 [MN181866.1_Europe_62 MN181860.1_Europe_68]

Hap_56: 7 [MN181865.1_Europe_63 MW503560.1_Europe_193 MN143279.1_Europe_219 MN182287.1_Europe_258 MH419815.1_Europe_294 HQ004961.1_Europe_390 MN182191.1_Europe_425]

Hap_57: 17 [MN181859.1_Europe_69 MN181858.1_Europe_70 MN181857.1_Europe_71 MN144000.1_Europe_209 MN182232.1_Europe_267 MH419853.1_Europe_292 MH419600.1_Europe_301 MH418828.1_Europe_332 MH418731.1_Europe_336 MN182217.1_Europe_357 MH420135.1_Europe_360 MH420314.1_Europe_362 MN182252.1_Europe_381 MW499236.1_Europe_477 MN182269.1_Europe_493 MN182271.1_Europe_495 MN181765.1_North_America_256]

Hap_58: 3 [MN181854.1_Europe_74 MH419554.1_Europe_304 MN182281.1_Europe_481]

Hap_59: 7 [MW503018.1_Europe_82 MN139054.1_Europe_109 MN138989.1_Europe_110 MN138915.1_Europe_111 MN138913.1_Europe_248 MN141453.1_Europe_403 MN182190.1_Europe_426]

Hap_60: 25 [MW502985.1_Europe_83 MN142540.1_Europe_92 MN182249.1_Europe_190 GU675894.1_Europe_452 MN182246.1_Europe_497 MN181899.1_Europe_502 MN181944.1_North_America_11 MN181756.1_North_America_24 MN181673.1_North_America_26 KM545461.1_North_America_34 KM540572.1_North_America_35 HQ583580.1_North_America_80 MN182331.1_North_America_81 MN182012.1_North_America_151 MN182006.1_North_America_156 MN181880.1_North_America_222 MN181772.1_North_America_247 MN181771.1_North_America_248 MN181767.1_North_America_252 MN181766.1_North_America_253 MN181763.1_North_America_254 MN181750.1_North_America_265 MN181725.1_North_America_288 MN181680.1_North_America_328 MN181624.1_North_America_379]

Hap_61: 1 [MN142384.1_Europe_95]

Hap_62: 2 [MN140313.1_Europe_103 MN182203.1_Europe_385]

Hap_63: 1 [MN139744.1_Europe_104]

Hap_64: 3 [MN182062.1_Europe_142 MH418714.1_Europe_164 MH419003.1_Europe_319]

Hap_65: 5 [MN182061.1_Europe_143 MN182056.1_Europe_148 MN182051.1_Europe_149 MN182055.1_Europe_153 MH419358.1_Europe_161]

Hap_66: 1 [MN182058.1_Europe_146]

Hap_67: 1 [MN181873.1_Europe_154]

Hap_68: 1 [MN181872.1_Europe_155]

Hap_69: 1 [MN181871.1_Europe_156]

Hap_70: 1 [MH418810.1_Europe_163]

Hap_71: 1 [HQ957208.1_Europe_166]

Hap_72: 2 [MW502266.1_Europe_178 MN143823.1_Europe_211]

Hap_73: 1 [MW500371.1_Europe_180]

Hap_74: 1 [MW499016.1_Europe_183]

Hap_75: 1 [MN182262.1_Europe_184]

Hap_76: 14 [MN182255.1_Europe_185 MN145219.1_Europe_201 MN143343.1_Europe_217 MN141684.1_Europe_229 MN140173.1_Europe_235 MN139868.1_Europe_238 MN139737.1_Europe_240 MH420266.1_Europe_275 MH420121.1_Europe_281 MH418966.1_Europe_320 MH418917.1_Europe_323 MH418860.1_Europe_328 MH418751.1_Europe_335 MH418718.1_Europe_337]

Hap_77: 2 [MN144699.1_Europe_204 MH419509.1_Europe_306]

Hap_78: 1 [MN144618.1_Europe_205]

Hap_79: 1 [MN143793.1_Europe_212]

Hap_80: 1 [MN142967.1_Europe_220]

Hap_81: 1 [MN139603.1_Europe_242]

Hap_82: 1 [MH420350.1_Europe_272]

Hap_83: 1 [MH420330.1_Europe_273]

Hap_84: 2 [MH420114.1_Europe_280 MH418495.1_Europe_346]

Hap_85: 2 [MH419871.1_Europe_290 MN182222.1_Europe_378]

Hap_86: 2 [MH419497.1_Europe_307 MH418752.1_Europe_334]

Hap_87: 1 [MH419334.1_Europe_310]

Hap_88: 1 [MH418677.1_Europe_338]

Hap_89: 1 [MH418663.1_Europe_339]

Hap_90: 1 [HQ004958.1_Europe_394]

Hap_91: 1 [HQ004953.1_Europe_397]

Hap_92: 1 [MN139365.1_Europe_411]

Hap_93: 2 [MN182236.1_Europe_418 MN182207.1_Europe_423]

Hap_94: 1 [MN182188.1_Europe_431]

Hap_95: 1 [GU669619.1_Europe_442]

Hap_96: 1 [JN827888.1_Europe_461]

Hap_97: 1 [MW502130.1_Europe_470]

Hap_98: 1 [MW501185.1_Europe_474]

Hap_99: 1 [MN182274.1_Europe_488]

Hap_100: 1 [MN182272.1_Europe_490]

Hap_101: 1 [MN182193.1_Europe_499]

Hap_102: 1 [MN181901.1_Europe_500]

Hap_103: 1 [GU673011.1_North_America_1]

Hap_104: 1 [GU673008.1_North_America_2]

Hap_105: 1 [GU673060.1_North_America_6]

Hap_106: 1 [GU672853.1_North_America_7]

Hap_107: 255 [MN182325.1_North_America_8 MN182010.1_North_America_9 MN181942.1_North_America_12 MN181939.1_North_America_15 MN181914.1_North_America_17 MN181761.1_North_America_19 MN181759.1_North_America_20 MN181760.1_North_America_21 MN181758.1_North_America_22 MN181757.1_North_America_23 MN181671.1_North_America_27 MN181647.1_North_America_28 MN181645.1_North_America_29 KM554514.1_North_America_30 KM549875.1_North_America_31 KM545769.1_North_America_33 KM541554.1_North_America_36 KM544400.1_North_America_37 KM545315.1_North_America_39 KM540520.1_North_America_40 JF841241.1_North_America_49 KJ165179.1_North_America_53 GU092010.1_North_America_54 GU092011.1_North_America_56 KT142082.1_North_America_58 KT138981.1_North_America_62 HQ583584.1_North_America_76 HQ583583.1_North_America_77 MN182329.1_North_America_82 MN182330.1_North_America_83 MN182327.1_North_America_85 MN182326.1_North_America_86 MN182324.1_North_America_87 MN182322.1_North_America_89 MN182320.1_North_America_91 MN182241.1_North_America_92 MN182166.1_North_America_93 MN182165.1_North_America_94 MN182164.1_North_America_95 MN182163.1_North_America_96 MN182161.1_North_America_98 MN182160.1_North_America_99 MN182159.1_North_America_100 MN182158.1_North_America_101 MN182157.1_North_America_102 MN182156.1_North_America_103 MN182155.1_North_America_104 MN182154.1_North_America_105 MN182153.1_North_America_106 MN182152.1_North_America_107 MN182151.1_North_America_108 MN182150.1_North_America_109 MN182149.1_North_America_110 MN182148.1_North_America_111 MN182147.1_North_America_112 MN182146.1_North_America_113 MN182145.1_North_America_114 MN182144.1_North_America_115 MN182143.1_North_America_116 MN182142.1_North_America_117 MN182141.1_North_America_118 MN182139.1_North_America_120 MN182138.1_North_America_121 MN182137.1_North_America_122 MN182136.1_North_America_123 MN182135.1_North_America_124 MN182134.1_North_America_125 MN182133.1_North_America_126 MN182132.1_North_America_127 MN182131.1_North_America_128 MN182127.1_North_America_132 MN182122.1_North_America_137 MN182121.1_North_America_138 MN182120.1_North_America_139 MN182047.1_North_America_140 MN182046.1_North_America_141 MN182045.1_North_America_142 MN182041.1_North_America_143 MN182040.1_North_America_144 MN182033.1_North_America_145 MN182023.1_North_America_146 MN182022.1_North_America_147 MN182021.1_North_America_148 MN182015.1_North_America_149 MN182013.1_North_America_150 MN182009.1_North_America_153 MN182007.1_North_America_155 MN182005.1_North_America_157 MN182003.1_North_America_159 MN182002.1_North_America_160 MN181986.1_North_America_164 MN181985.1_North_America_165 MN181984.1_North_America_166 MN181983.1_North_America_167 MN181982.1_North_America_168 MN181981.1_North_America_169 MN181980.1_North_America_170 MN181979.1_North_America_171 MN181978.1_North_America_172 MN181977.1_North_America_173 MN181976.1_North_America_174 MN181974.1_North_America_175 MN181975.1_North_America_176 MN181973.1_North_America_177 MN181971.1_North_America_179 MN181946.1_North_America_180 MN181943.1_North_America_181 MN181937.1_North_America_182 MN181936.1_North_America_183 MN181935.1_North_America_184 MN181934.1_North_America_185 MN181933.1_North_America_186 MN181932.1_North_America_187 MN181931.1_North_America_188 MN181930.1_North_America_189 MN181929.1_North_America_190 MN181928.1_North_America_191 MN181927.1_North_America_192 MN181926.1_North_America_193 MN181925.1_North_America_194 MN181924.1_North_America_195 MN181923.1_North_America_196 MN181922.1_North_America_197 MN181921.1_North_America_198 MN181920.1_North_America_199 MN181919.1_North_America_200 MN181918.1_North_America_201 MN181917.1_North_America_202 MN181916.1_North_America_203 MN181915.1_North_America_204 MN181913.1_North_America_205 MN181912.1_North_America_206 MN181911.1_North_America_207 MN181909.1_North_America_209 MN181908.1_North_America_210 MN181907.1_North_America_211 MN181906.1_North_America_212 MN181903.1_North_America_213 MN181893.1_North_America_217 MN181892.1_North_America_218 MN181883.1_North_America_219 MN181882.1_North_America_220 MN181881.1_North_America_221 MN181879.1_North_America_223 MN181878.1_North_America_224 MN181877.1_North_America_225 MN181876.1_North_America_226 MN181792.1_North_America_227 MN181790.1_North_America_229 MN181789.1_North_America_230 MN181788.1_North_America_231 MN181787.1_North_America_232 MN181786.1_North_America_233 MN181785.1_North_America_234 MN181784.1_North_America_235 MN181783.1_North_America_236 MN181782.1_North_America_237 MN181781.1_North_America_238 MN181780.1_North_America_239 MN181779.1_North_America_240 MN181777.1_North_America_242 MN181776.1_North_America_243 MN181774.1_North_America_245 MN181773.1_North_America_246 MN181768.1_North_America_251 MN181764.1_North_America_255 MN181754.1_North_America_257 MN181753.1_North_America_258 MN181751.1_North_America_260 MN181746.1_North_America_261 MN181748.1_North_America_263 MN181749.1_North_America_264 MN181744.1_North_America_267 MN181743.1_North_America_268 MN181742.1_North_America_269 MN181736.1_North_America_271 MN181737.1_North_America_272 MN181738.1_North_America_273 MN181739.1_North_America_274 MN181735.1_North_America_276 MN181734.1_North_America_277 MN181727.1_North_America_282 MN181723.1_North_America_286 MN181722.1_North_America_289 MN181721.1_North_America_290 MN181720.1_North_America_291 MN181719.1_North_America_292 MN181717.1_North_America_294 MN181715.1_North_America_296 MN181714.1_North_America_297 MN181713.1_North_America_298 MN181712.1_North_America_299 MN181711.1_North_America_300 MN181706.1_North_America_301 MN181707.1_North_America_302 MN181708.1_North_America_303 MN181709.1_North_America_304 MN181710.1_North_America_305 MN181701.1_North_America_306 MN181703.1_North_America_308 MN181704.1_North_America_309 MN181705.1_North_America_310 MN181700.1_North_America_311 MN181699.1_North_America_312 MN181698.1_North_America_313 MN181697.1_North_America_314 MN181689.1_North_America_318 MN181687.1_North_America_320 MN181682.1_North_America_323 MN181676.1_North_America_325 MN181678.1_North_America_327 MN181674.1_North_America_331 MN181675.1_North_America_332 MN181670.1_North_America_333 MN181669.1_North_America_334 MN181668.1_North_America_335 MN181667.1_North_America_336 MN181666.1_North_America_337 MN181661.1_North_America_338 MN181662.1_North_America_339 MN181663.1_North_America_340 MN181664.1_North_America_341 MN181656.1_North_America_343 MN181657.1_North_America_344 MN181658.1_North_America_345 MN181659.1_North_America_346 MN181660.1_North_America_347 MN181651.1_North_America_348 MN181653.1_North_America_350 MN181654.1_North_America_351 MN181655.1_North_America_352 MN181646.1_North_America_353 MN181648.1_North_America_354 MN181649.1_North_America_355 MN181650.1_North_America_356 MN181644.1_North_America_357 MN181643.1_North_America_358 MN181641.1_North_America_360 MN181636.1_North_America_361 MN181637.1_North_America_362 MN181638.1_North_America_363 MN181640.1_North_America_364 MN181639.1_North_America_365 MN181635.1_North_America_366 MN181632.1_North_America_368 MN181634.1_North_America_369 MN181626.1_North_America_371 MN181627.1_North_America_372 MN181628.1_North_America_373 MN181629.1_North_America_374 MN181630.1_North_America_375 MN181621.1_North_America_376 MN181622.1_North_America_377 MN181623.1_North_America_378 MN181625.1_North_America_380]

Hap_108: 1 [MN181945.1_North_America_10]

Hap_109: 1 [MN181941.1_North_America_13]

Hap_110: 5 [MN181940.1_North_America_14 MN181910.1_North_America_208 MN181791.1_North_America_228 MN181745.1_North_America_266 MN181633.1_North_America_370]

Hap_111: 1 [MN182004.1_North_America_158]

Hap_112: 1 [MN181995.1_North_America_162]

Hap_113: 1 [MN181994.1_North_America_163]

Hap_114: 1 [MN181897.1_North_America_216]

Hap_115: 1 [MN181775.1_North_America_244]

Hap_116: 1 [JF283398.1_North_America_381]

Hap_117: 1 [MN181840.1_Oceania_38]
