# Supplementary material for: Phylogeography and genetic diversity of Pieris rapae across continents
Source: PLoS One. 2026 Jul 24;21(7):e0354257. doi: 10.1371/journal.pone.0354257 (PMC13399322; doi:10.1371/journal.pone.0354257)
Supplement: Supplementary file 3 [file pone.0354257.s001.odt]

S1 Table. NCBI accession information for 1133 mitochondrial COI sequences of Pieris rapae from the five continents. 

Africa

| Sl. No | Accession | GPS | Country |
| --- | --- | --- | --- |
| 1 | MN182318.1 | 36°37'48.0"N 4°36'00.0"E | Algeria |
| 2 | MN182311.1 | 36°54'36.0"N 7°45'36.0"E |
| 3 | MN182312.1 | 35°51'36.0"N 4°45'00.0"E |
| 4 | MN182313.1 | 35°45'00.0"N 4°33'00.0"E |
| 5 | MN182314.1 | 35°11'24.0"N 1°07'48.0"E |
| 6 | MN182307.1 | 35°19'48.0"N 4°18'00.0"E |
| 7 | MN182308.1 | 35°19'48.0"N 4°18'00.0"E |
| 8 | MN182309.1 | 36°35'24.0"N 4°36'36.0"E |
| 9 | MH420349.1 | 35°11'24.0"N 1°07'48.0"E |
| 10 | MH418536.1 | 36°26'45.6"N 4°06'46.8"E |
| 11 | MH418735.1 | 35°11'24.0"N 1°07'48.0"E |
| 12 | MH418996.1 | 35°11'24.0"N 1°07'48.0"E |
| 13 | MH419526.1 | 35°13'12.0"N 4°11'24.0"E |
| 14 | MH419632.1 | 35°13'12.0"N 4°11'24.0"E |
| 15 | MH419399.1 | 35°42'36.0"N 4°31'12.0"E |
| 16 | MH419498.1 | 35°13'12.0"N 4°11'24.0"E |
| 17 | MH419165.1 | 35°13'12.0"N 4°11'24.0"E |
| 18 | MH419177.1 | 35°13'12.0"N 4°11'24.0"E |
| 19 | MH418626.1 | 35°16'48.0"N 0°55'12.0"E |
| 20 | MN182296.1 | 33°39'36.0"N 4°51'00.0"W | Morocco |
| 21 | MN182297.1 | 33°39'36.0"N 4°51'00.0"W |
| 22 | MN182298.1 | 33°39'36.0"N 4°51'00.0"W |
| 23 | MN182299.1 | 33°39'36.0"N 4°51'00.0"W |
| 24 | MN182300.1 | 34°07'12.0"N 4°02'24.0"W |
| 25 | MN182303.1 | 34°07'12.0"N 4°02'24.0"W |
| 26 | MN182302.1 | 35°42'00.0"N 5°37'48.0"W |
| 27 | MN182301.1 | 35°42'00.0"N 5°37'48.0"W |
| 28 | MN182304.1 | 35°42'00.0"N 5°37'48.0"W |
| 29 | MN182315.1 | 35°55'48.0"N 8°32'24.0"E | Tunisia |
| 30 | MN182316.1 | 37°09'36.0"N 10°06'36.0"E |
| 31 | MN182317.1 | 37°09'36.0"N 10°06'36.0"E |
| 32 | MN182310.1 | 37°09'36.0"N 10°06'36.0"E |
| 33 | MN182305.1 | 33°56'31.2"N 8°10'22.8"E |
| 34 | MN182306.1 | 33°56'31.2"N 8°10'22.8"E |
| 35 | MH419260.1 | 33°56'31.2"N 8°10'22.8"E |
| 36 | MH419423.1 | 35°03'10.8"N 9°15'25.2"E |
| 37 | MH419186.1 | 35°03'10.8"N 9°15'25.2"E |
| 38 | MH419959.1 | 35°03'10.8"N 9°15'25.2"E |
| 39 | MH420018.1 | 35°03'10.8"N 9°15'25.2"E |
| 40 | MH419503.1 | 33°24'25.2"N 9°00'28.8"E |
| 41 | MH419253.1 | 33°24'25.2"N 9°00'28.8"E |
| 42 | MH419320.1 | 33°24'25.2"N 9°00'28.8"E |
| 43 | MH418702.1 | 35°25'30.0"N 9°34'15.6"E |
| 44 | MH418814.1 | 35°25'30.0"N 9°34'15.6"E |
| 45 | MH419084.1 | 36°46'55.2"N 8°45'03.6"E |
| 46 | MH419137.1 | 36°46'55.2"N 8°45'03.6"E |

Asia

| Sl. No | Accession | GPS | Country |
| --- | --- | --- | --- |
| 1 | MN182095.1 | 27°56'24.0"N 108°37'12.0"E | China |
| 2 | MN182094.1 | 27°56'24.0"N 108°37'12.0"E |
| 3 | MN182093.1 | 27°56'24.0"N 108°37'12.0"E |
| 4 | MN182092.1 | 27°56'24.0"N 108°37'12.0"E |
| 5 | MN182091.1 | 27°56'24.0"N 108°37'12.0"E |
| 6 | MN182090.1 | 27°56'24.0"N 108°37'12.0"E |
| 7 | MN182089.1 | 31°29'24.0"N 98°13'12.0"E |
| 8 | MN182088.1 | 31°29'24.0"N 98°13'12.0"E |
| 9 | MN182087.1 | 31°29'24.0"N 98°13'12.0"E |
| 10 | MN182086.1 | 31°29'24.0"N 98°13'12.0"E |
| 11 | MN182085.1 | 31°29'24.0"N 98°13'12.0"E |
| 12 | MN182084.1 | 31°29'24.0"N 98°13'12.0"E |
| 13 | MN182083.1 | 31°29'24.0"N 98°13'12.0"E |
| 14 | MN182082.1 | 31°29'24.0"N 98°13'12.0"E |
| 15 | MN182081.1 | 31°29'24.0"N 98°13'12.0"E |
| 16 | MN182080.1 | 31°29'24.0"N 98°13'12.0"E |
| 17 | MN182079.1 | 43°49'12.0"N 127°24'36.0"E |
| 18 | MN182078.1 | 31°08'24.0"N 104°24'00.0"E |
| 19 | MN182077.1 | 36°45'36.0"N 118°00'00.0"E |
| 20 | MN182076.1 | 36°15'36.0"N 137°58'12.0"E | Japan |
| 21 | MN182074.1 | 37°58'12.0"N 139°19'48.0"E |
| 22 | MN182075.1 | 37°58'12.0"N 139°19'48.0"E |
| 23 | MN182069.1 | 37°58'12.0"N 139°19'48.0"E |
| 24 | MN182068.1 | 37°58'12.0"N 139°19'48.0"E |
| 25 | MN182067.1 | 36°08'24.0"N 140°07'12.0"E |
| 26 | MN182066.1 | 36°08'24.0"N 140°07'12.0"E |
| 27 | MN182065.1 | 36°08'24.0"N 140°07'12.0"E |
| 28 | MN181886.1 | 36°08'24.0"N 140°07'12.0"E |
| 29 | MN181885.1 | 36°08'24.0"N 140°07'12.0"E |
| 30 | MN181884.1 | 36°08'24.0"N 140°07'12.0"E |
| 31 | MN182072.1 | 36°27'00.0"N 126°52'48.0"E | South Korea |
| 32 | MN182071.1 | 36°27'00.0"N 126°52'48.0"E |
| 33 | MN182070.1 | 36°27'00.0"N 126°52'48.0"E |
| 34 | MN181970.1 | 36°36'36.0"N 129°23'24.0"E |
| 35 | MN181969.1 | 37°10'12.0"N 128°22'12.0"E |
| 36 | MN181968.1 | 37°10'12.0"N 128°22'12.0"E |
| 37 | MN181967.1 | 33°25'48.0"N 126°28'12.0"E |
| 38 | MN181966.1 | 33°25'48.0"N 126°28'12.0"E |
| 39 | MN181965.1 | 33°28'48.0"N 126°42'36.0"E |
| 40 | GU696024.1 | 36°37'40.8"N 127°27'14.4"E |
| 41 | EU105305.1 | 33°23'32.4"N 126°24'58.4"E |
| 42 | EU105304.1 | 33°23'32.4"N 126°24'58.4"E |
| 43 | EU105303.1 | 33°23'32.4"N 126°24'58.4"E |
| 44 | EU105302.1 | 33°23'32.4"N 126°24'58.4"E |
| 45 | EU105297.1 | 33°23'32.4"N 126°24'58.4"E |
| 46 | EU105298.1 | 33°23'32.4"N 126°24'58.4"E |
| 47 | EU105299.1 | 33°23'32.4"N 126°24'58.4"E |
| 48 | EU105300.1 | 33°23'32.4"N 126°24'58.4"E |
| 49 | EU105301.1 | 33°23'32.4"N 126°24'58.4"E |
| 50 | EU105296.1 | 33°23'32.4"N 126°24'58.4"E |
| 51 | EU105295.1 | 33°23'32.4"N 126°24'58.4"E |
| 52 | EU105294.1 | 33°23'32.4"N 126°24'58.4"E |
| 53 | EU105293.1 | 33°23'32.4"N 126°24'58.4"E |
| 54 | EU105292.1 | 33°23'32.4"N 126°24'58.4"E |
| 55 | EU105291.1 | 33°23'32.4"N 126°24'58.4"E |
| 56 | EU105290.1 | 33°23'32.4"N 126°24'58.4"E |
| 57 | EU105289.1 | 34°50'12.6"N 127°52'18.8"E |
| 58 | EU105288.1 | 34°50'12.6"N 127°52'18.8"E |
| 59 | EU105287.1 | 34°50'12.6"N 127°52'18.8"E |
| 60 | EU105286.1 | 34°50'12.6"N 127°52'18.8"E |
| 61 | EU105285.1 | 34°50'12.6"N 127°52'18.8"E |
| 62 | EU105284.1 | 34°50'12.6"N 127°52'18.8"E |
| 63 | EU105283.1 | 34°50'12.6"N 127°52'18.8"E |
| 64 | EU105282.1 | 34°50'12.6"N 127°52'18.8"E |
| 65 | EU105277.1 | 34°50'12.6"N 127°52'18.8"E |
| 66 | EU105278.1 | 34°50'12.6"N 127°52'18.8"E |
| 67 | EU105279.1 | 34°50'12.6"N 127°52'18.8"E |
| 68 | EU105280.1 | 34°50'12.6"N 127°52'18.8"E |
| 69 | EU105281.1 | 34°50'12.6"N 127°52'18.8"E |
| 70 | EU105272.1 | 34°50'12.6"N 127°52'18.8"E |
| 71 | EU105273.1 | 34°50'12.6"N 127°52'18.8"E |
| 72 | EU105274.1 | 34°50'12.6"N 127°52'18.8"E |
| 73 | EU105275.1 | 34°50'12.6"N 127°52'18.8"E |
| 74 | EU105276.1 | 34°50'12.6"N 127°52'18.8"E |
| 75 | EU105267.1 | 34°50'12.6"N 127°52'18.8"E |
| 76 | EU105268.1 | 34°50'12.6"N 127°52'18.8"E |
| 77 | EU105269.1 | 34°50'12.6"N 127°52'18.8"E |
| 78 | EU105270.1 | 34°50'12.6"N 127°52'18.8"E |
| 79 | EU105271.1 | 34°50'12.6"N 127°52'18.8"E |
| 80 | EU105262.1 | 35°16'42.6"N 127°17'52.4"E |
| 81 | EU105263.1 | 35°16'42.6"N 127°17'52.4"E |
| 82 | EU105264.1 | 35°16'42.6"N 127°17'52.4"E |
| 83 | EU105265.1 | 35°16'42.6"N 127°17'52.4"E |
| 84 | EU105266.1 | 35°16'42.6"N 127°17'52.4"E |
| 85 | EU105261.1 | 35°16'42.6"N 127°17'52.4"E |
| 86 | EU105260.1 | 35°16'42.6"N 127°17'52.4"E |
| 87 | EU105259.1 | 35°16'42.6"N 127°17'52.4"E |
| 88 | EU105258.1 | 35°16'42.6"N 127°17'52.4"E |
| 89 | EU105257.1 | 35°16'42.6"N 127°17'52.4"E |
| 90 | EU105252.1 | 35°08'38.5"N 126°59'20.3"E |
| 91 | EU105253.1 | 35°08'38.5"N 126°59'20.3"E |
| 92 | EU105254.1 | 35°08'38.5"N 126°59'20.3"E |
| 93 | EU105255.1 | 35°08'38.5"N 126°59'20.3"E |
| 94 | EU105256.1 | 35°08'38.5"N 126°59'20.3"E |
| 95 | EU105247.1 | 35°08'38.5"N 126°59'20.3"E |
| 96 | EU105248.1 | 35°08'38.5"N 126°59'20.3"E |
| 97 | EU105249.1 | 35°08'38.5"N 126°59'20.3"E |
| 98 | EU105250.1 | 35°08'38.5"N 126°59'20.3"E |
| 99 | EU105251.1 | 35°08'38.5"N 126°59'20.3"E |
| 100 | EU105242.1 | 35°08'38.5"N 126°59'20.3"E |
| 101 | EU105243.1 | 35°08'38.5"N 126°59'20.3"E |
| 102 | EU105244.1 | 35°08'38.5"N 126°59'20.3"E |
| 103 | EU105245.1 | 35°08'38.5"N 126°59'20.3"E |
| 104 | EU105246.1 | 35°08'38.5"N 126°59'20.3"E |
| 105 | EU105232.1 | 35°15'25.2"N 127°27'26.2"E |
| 106 | EU105233.1 | 35°15'25.2"N 127°27'26.2"E |
| 107 | EU105234.1 | 35°15'25.2"N 127°27'26.2"E |
| 108 | EU105235.1 | 35°15'25.2"N 127°27'26.2"E |
| 109 | EU105236.1 | 35°15'25.2"N 127°27'26.2"E |
| 110 | EU105237.1 | 35°15'25.2"N 127°27'26.2"E |
| 111 | EU105238.1 | 35°15'25.2"N 127°27'26.2"E |
| 112 | EU105239.1 | 35°15'25.2"N 127°27'26.2"E |
| 113 | EU105240.1 | 35°15'25.2"N 127°27'26.2"E |
| 114 | EU105241.1 | 35°08'01.9"N 126°59'21.4"E |
| 115 | EU105222.1 | 36°39'03.7"N 128°27'51.4"E |
| 116 | EU105223.1 | 36°39'03.7"N 128°27'51.4"E |
| 117 | EU105224.1 | 36°39'03.7"N 128°27'51.4"E |
| 118 | EU105225.1 | 36°39'03.7"N 128°27'51.4"E |
| 119 | EU105226.1 | 36°39'03.7"N 128°27'51.4"E |
| 120 | EU105227.1 | 36°39'03.7"N 128°27'51.4"E |
| 121 | EU105228.1 | 35°15'25.2"N 127°27'26.2"E |
| 122 | EU105229.1 | 35°15'25.2"N 127°27'26.2"E |
| 123 | EU105230.1 | 35°15'25.2"N 127°27'26.2"E |
| 124 | EU105231.1 | 35°15'25.2"N 127°27'26.2"E |
| 125 | EU105217.1 | 37°14'28.4"N 126°34'43.1"E |
| 126 | EU105218.1 | 37°14'28.4"N 126°34'43.1"E |
| 127 | EU105219.1 | 37°14'28.4"N 126°34'43.1"E |
| 128 | EU105220.1 | 37°14'28.4"N 126°34'43.1"E |
| 129 | EU105221.1 | 37°14'28.4"N 126°34'43.1"E |
| 130 | EU105213.1 | 37°14'28.4"N 126°34'43.1"E |
| 131 | EU105214.1 | 37°14'28.4"N 126°34'43.1"E |
| 132 | EU105215.1 | 37°14'28.4"N 126°34'43.1"E |
| 133 | EU105216.1 | 37°14'28.4"N 126°34'43.1"E |
| 134 | JX445952.1 | 37°14'28.4"N 126°34'43.1"E |
| 135 | JX242475.1 | 37°14'28.4"N 126°34'43.1"E |
| 136 | JX242474.1 | 37°14'28.4"N 126°34'43.1"E |
| 137 | MN182106.1 | 24°57'36.0"N 121°27'00.0"E | Taiwan |
| 138 | MN182105.1 | 24°57'36.0"N 121°27'00.0"E |
| 139 | MN182104.1 | 24°57'36.0"N 121°27'00.0"E |
| 140 | MN182103.1 | 24°57'36.0"N 121°27'00.0"E |
| 141 | MN182102.1 | 24°57'36.0"N 121°27'00.0"E |
| 142 | MN182101.1 | 24°57'36.0"N 121°27'00.0"E |
| 143 | MN182100.1 | 24°57'36.0"N 121°27'00.0"E |
| 144 | MN182099.1 | 25°01'12.0"N 121°31'48.0"E |
| 145 | MN182098.1 | 25°01'12.0"N 121°31'48.0"E |
| 146 | MN182097.1 | 25°01'12.0"N 121°31'48.0"E |
| 147 | MN182096.1 | 25°01'12.0"N 121°31'48.0"E |
| 148 | MN182037.1 | 24°57'36.0"N 121°27'00.0"E |
| 149 | MN182036.1 | 24°57'36.0"N 121°27'00.0"E |
| 150 | MN182035.1 | 24°57'36.0"N 121°27'00.0"E |
| 151 | MN182034.1 | 25°01'12.0"N 121°31'48.0"E |
| 152 | MN182032.1 | 24°50'24.0"N 121°16'12.0"E |
| 153 | MN182031.1 | 24°57'36.0"N 121°27'00.0"E |
| 154 | MN182030.1 | 24°57'36.0"N 121°27'00.0"E |
| 155 | MN182029.1 | 25°01'12.0"N 121°31'48.0"E |
| 156 | MN182028.1 | 25°01'12.0"N 121°31'48.0"E |
| 157 | MN182027.1 | 25°01'12.0"N 121°31'48.0"E |
| 158 | MN182026.1 | 25°01'12.0"N 121°31'48.0"E |
| 159 | MN182025.1 | 25°01'12.0"N 121°31'48.0"E |
| 160 | MN182024.1 | 25°01'12.0"N 121°31'48.0"E |

Europe

| Sl. No | Accession | GPS | Country |
| --- | --- | --- | --- |
| 1 | MW502246.1 | 46°47'25.8"N 12°51'42.8"E | Austria |
| 2 | MW501076.1 | 47°35'12.8"N 13°33'37.9"E |
| 3 | MN145333.1 | 47°25'44.4"N 10°37'30.0"E |
| 4 | MN144447.1 | 47°49'30.0"N 16°02'37.0"E |
| 5 | MN144390.1 | 47°28'01.2"N 13°49'58.8"E |
| 6 | MN144242.1 | 47°49'30.0"N 16°02'37.0"E |
| 7 | MN144127.1 | 47°11'24.0"N 11°06'00.0"E |
| 8 | MN142981.1 | 47°24'36.0"N 14°12'36.0"E |
| 9 | MN139941.1 | 47°48'20.2"N 16°38'11.0"E |
| 10 | MN139621.1 | 47°43'01.2"N 15°46'01.2"E |
| 11 | MN139482.1 | 46°36'19.8"N 13°41'20.0"E |
| 12 | KM573655.1 | 47°08'16.8"N 10°11'49.2"E |
| 13 | KC462818.1 | 48°14'14.0"N 16°12'11.7"E |
| 14 | KC462817.1 | 48°14'14.0"N 16°12'11.7"E |
| 15 | KC462816.1 | 48°14'14.0"N 16°12'11.7"E |
| 16 | OR891202.1 | 48°50'49.2"N 15°58'22.8"E | Czech Republic |
| 17 | OR891201.1 | 48°50'49.2"N 15°58'22.8"E |
| 18 | OR891200.1 | 48°50'49.2"N 15°58'22.8"E |
| 19 | OR891199.1 | 48°50'49.2"N 15°58'22.8"E |
| 20 | OR891198.1 | 48°50'49.2"N 15°58'22.8"E |
| 21 | OR891197.1 | 48°58'26.4"N 14°28'26.4"E |
| 22 | OR891196.1 | 48°58'19.2"N 14°31'48.0"E |
| 23 | OR891195.1 | 49°26'06.0"N 12°44'31.2"E |
| 24 | OR891194.1 | 49°26'06.0"N 12°44'31.2"E |
| 25 | OR891193.1 | 48°58'19.2"N 14°31'48.0"E |
| 26 | OR891192.1 | 48°58'30.0"N 14°26'38.4"E |
| 27 | OR891191.1 | 48°58'30.0"N 14°26'38.4"E |
| 28 | OR891190.1 | 48°58'30.0"N 14°26'38.4"E |
| 29 | OR891189.1 | 48°58'30.0"N 14°26'38.4"E |
| 30 | OR891188.1 | 48°58'30.0"N 14°26'38.4"E |
| 31 | OR891187.1 | 48°58'30.0"N 14°26'38.4"E |
| 32 | OR891185.1 | 48°58'30.0"N 14°26'38.4"E |
| 33 | OR891184.1 | 48°58'30.0"N 14°26'38.4"E |
| 34 | OR891183.1 | 48°57'25.2"N 14°32'34.8"E |
| 35 | OR891182.1 | 49°59'49.2"N 14°48'46.8"E |
| 36 | OR891181.1 | 48°58'19.2"N 14°31'48.0"E |
| 37 | OR891180.1 | 49°26'06.0"N 12°44'31.2"E |
| 38 | MW501122.1 | 48°48'25.9"N 15°59'25.1"E |
| 39 | MN182184.1 | 49°30'00.0"N 17°00'00.0"E |
| 40 | MN182183.1 | 49°30'00.0"N 17°00'00.0"E |
| 41 | MN182182.1 | 49°30'00.0"N 17°00'00.0"E |
| 42 | MN182181.1 | 49°30'00.0"N 17°00'00.0"E |
| 43 | MN182180.1 | 49°30'00.0"N 17°00'00.0"E |
| 44 | MN182179.1 | 49°30'00.0"N 17°00'00.0"E |
| 45 | MN182178.1 | 49°30'00.0"N 17°00'00.0"E |
| 46 | MN182177.1 | 49°30'00.0"N 17°00'00.0"E |
| 47 | MN182176.1 | 49°30'00.0"N 17°00'00.0"E |
| 48 | MN182175.1 | 49°30'00.0"N 17°00'00.0"E |
| 49 | MN182174.1 | 49°30'00.0"N 17°00'00.0"E |
| 50 | MN182173.1 | 49°30'00.0"N 17°00'00.0"E |
| 51 | MN182172.1 | 49°30'00.0"N 17°00'00.0"E |
| 52 | MN182171.1 | 49°30'00.0"N 17°00'00.0"E |
| 53 | MN182170.1 | 49°30'00.0"N 17°00'00.0"E |
| 54 | MN182169.1 | 49°30'00.0"N 17°00'00.0"E |
| 55 | MN182168.1 | 49°30'00.0"N 17°00'00.0"E |
| 56 | MN182167.1 | 49°30'00.0"N 17°00'00.0"E |
| 57 | MN181870.1 | 49°30'00.0"N 17°00'00.0"E |
| 58 | MN181869.1 | 49°30'00.0"N 17°00'00.0"E |
| 59 | MN181868.1 | 49°30'00.0"N 17°00'00.0"E |
| 60 | MN181867.1 | 49°30'00.0"N 17°00'00.0"E |
| 61 | MN181866.1 | 49°30'00.0"N 17°00'00.0"E |
| 62 | MN181865.1 | 49°30'00.0"N 17°00'00.0"E |
| 63 | MN181864.1 | 49°30'00.0"N 17°00'00.0"E |
| 64 | MN181863.1 | 49°30'00.0"N 17°00'00.0"E |
| 65 | MN181862.1 | 49°30'00.0"N 17°00'00.0"E |
| 66 | MN181861.1 | 49°30'00.0"N 17°00'00.0"E |
| 67 | MN181860.1 | 49°30'00.0"N 17°00'00.0"E |
| 68 | MN181859.1 | 49°30'00.0"N 17°00'00.0"E |
| 69 | MN181858.1 | 49°30'00.0"N 17°00'00.0"E |
| 70 | MN181857.1 | 49°30'00.0"N 17°00'00.0"E |
| 71 | MN181856.1 | 49°30'00.0"N 17°00'00.0"E |
| 72 | MN181855.1 | 49°30'00.0"N 17°00'00.0"E |
| 73 | MN181854.1 | 49°30'00.0"N 17°00'00.0"E |
| 74 | MN181853.1 | 49°30'00.0"N 17°00'00.0"E |
| 75 | MN181852.1 | 49°30'00.0"N 17°00'00.0"E |
| 76 | MN181851.1 | 49°30'00.0"N 17°00'00.0"E |
| 77 | MN181850.1 | 49°30'00.0"N 17°00'00.0"E |
| 78 | MN181849.1 | 49°30'00.0"N 17°00'00.0"E |
| 79 | MN181848.1 | 49°30'00.0"N 17°00'00.0"E |
| 80 | MN181847.1 | 49°30'00.0"N 17°00'00.0"E |
| 81 | MW503018.1 | 42°43'39.0"N 1°49'46.9"E | France |
| 82 | MW502985.1 | 46°22'25.0"N 5°55'33.9"E |
| 83 | MW501754.1 | 49°02'57.1"N 7°25'35.7"E |
| 84 | MN144711.1 | 47°53'06.0"N 3°13'40.8"E |
| 85 | MN144671.1 | 42°33'21.6"N 1°48'54.0"E |
| 86 | MN143517.1 | 50°17'02.4"N 2°57'28.8"E |
| 87 | MN143210.1 | 46°17'56.4"N 1°05'49.2"W |
| 88 | MN142999.1 | 44°00'46.8"N 3°50'56.4"E |
| 89 | MN142903.1 | 47°18'36.0"N 4°05'24.0"E |
| 90 | MN142773.1 | 43°01'12.0"N 6°26'02.4"E |
| 91 | MN142540.1 | 46°06'57.6"N 5°37'40.8"E |
| 92 | MN142492.1 | 50°42'07.2"N 2°14'02.4"E |
| 93 | MN142439.1 | 44°04'08.4"N 7°12'28.8"E |
| 94 | MN142384.1 | 44°42'00.0"N 5°31'30.0"E |
| 95 | MN142102.1 | 48°43'37.2"N 2°01'22.8"E |
| 96 | MN141970.1 | 50°59'06.0"N 1°57'25.2"E |
| 97 | MN141703.1 | 42°51'14.4"N 2°47'24.0"E |
| 98 | MN141637.1 | 44°04'55.2"N 6°11'16.8"E |
| 99 | MN141407.1 | 43°33'14.4"N 5°43'48.0"E |
| 100 | MN141067.1 | 43°35'45.6"N 5°10'01.2"E |

	

|  | Accession | GPS | Country |
| --- | --- | --- | --- |
| 101 | MN141075.1 | 48°29'20.4"N 2°29'49.2"E | France |
| 102 | MN140313.1 | 50°59'06.0"N 1°57'25.2"E |
| 103 | MN139744.1 | 47°15'39.6"N 4°34'12.0"E |
| 104 | MN139623.1 | 44°31'12.0"N 0°36'00.0"W |
| 105 | MN139564.1 | 45°52'08.4"N 6°40'51.6"E |
| 106 | MN139542.1 | 43°33'14.4"N 5°43'48.0"E |
| 107 | MN139131.1 | 44°35'24.0"N 0°34'48.0"W |
| 108 | MN139054.1 | 47°39'10.8"N 3°45'28.8"E |
| 109 | MN138989.1 | 43°02'52.8"N 2°52'40.8"E |
| 110 | MN138915.1 | 49°04'37.2"N 2°14'49.2"E |
| 111 | MN138806.1 | 43°19'51.6"N 5°41'20.4"E |
| 112 | MN138788.1 | 43°30'57.6"N 3°40'04.8"E |
| 113 | MN138553.1 | 43°01'12.0"N 6°26'02.4"E |
| 114 | MN182319.1 | 46°25'48.0"N 1°01'48.0"W |
| 115 | MN182266.1 | 44°12'00.0"N 7°04'12.0"E |
| 116 | MN182265.1 | 42°55'48.0"N 2°57'36.0"E |
| 117 | MN182242.1 | 47°39'10.8"N 3°45'28.8"E |
| 118 | MN182240.1 | 43°02'52.8"N 2°52'40.8"E |
| 119 | MN182239.1 | 49°04'37.2"N 2°14'49.2"E |
| 120 | MN182212.1 | 43°19'51.6"N 5°41'20.4"E |
| 121 | MN182208.1 | 43°30'57.6"N 3°40'04.8"E |
| 122 | MN182198.1 | 43°01'12.0"N 6°26'02.4"E |
| 123 | MH420062.1 | 46°25'48.0"N 1°01'48.0"W |
| 124 | MH419923.1 | 44°12'00.0"N 7°04'12.0"E |
| 125 | MH419525.1 | 42°55'48.0"N 2°57'36.0"E |
| 126 | MH419453.1 | 43°01'12.0"N 6°26'02.4"E |
| 127 | MH419376.1 | 46°25'48.0"N 1°01'48.0"W |
| 128 | MH419326.1 | 44°12'00.0"N 7°04'12.0"E |
| 129 | MH418874.1 | 42°55'48.0"N 2°57'36.0"E |
| 130 | KX041897.1 | 49°29'34.8"N 1°28'55.2"E |
| 131 | MN182064.1 | 42°00'00.0"N 45°34'12.0"E | Georgia |
| 132 | MN182063.1 | 42°00'00.0"N 45°34'12.0"E |
| 133 | MN182062.1 | 42°00'00.0"N 45°34'12.0"E |
| 134 | MN182061.1 | 42°00'00.0"N 45°34'12.0"E |
| 135 | MN182060.1 | 41°36'00.0"N 41°35'24.0"E |
| 136 | MN182059.1 | 42°00'00.0"N 45°34'12.0"E |
| 137 | MN182058.1 | 42°00'00.0"N 45°34'12.0"E |
| 138 | MN182057.1 | 42°00'00.0"N 45°34'12.0"E |
| 139 | MN182056.1 | 42°00'00.0"N 45°34'12.0"E |
| 140 | MN182051.1 | 42°16'48.0"N 41°40'48.0"E |
| 141 | MN182052.1 | 42°00'00.0"N 45°34'12.0"E |
| 142 | MN182053.1 | 42°16'48.0"N 41°40'48.0"E |
| 143 | MN182054.1 | 42°16'48.0"N 41°40'48.0"E |
| 144 | MN182055.1 | 42°16'48.0"N 41°40'48.0"E |
| 145 | MN181873.1 | 42°12'00.0"N 41°57'00.0"E |
| 146 | MN181872.1 | 42°12'00.0"N 41°57'00.0"E |
| 147 | MN181871.1 | 42°16'48.0"N 41°40'48.0"E |
| 148 | MH420231.1 | 49°42'17.3"N 7°20'51.0"E | Germany |
| 149 | MH419958.1 | 51°20'19.0"N 13°56'44.9"E |
| 150 | MH419873.1 | 49°59'06.0"N 9°46'15.6"E |
| 151 | MH419592.1 | 49°42'17.3"N 7°20'51.0"E |
| 152 | MH419358.1 | 51°20'19.0"N 13°56'44.9"E |
| 153 | MH419172.1 | 49°59'06.0"N 9°46'15.6"E |
| 154 | MH418810.1 | 50°40'18.8"N 11°08'30.1"E |
| 155 | MH418714.1 | 50°40'18.8"N 11°08'30.1"E |
| 156 | HQ957208.1 | 48°52'51.6"N 12°27'21.6"E |
| 157 | MW503297.1 | 40°57'39.6"N 26°03'57.6"E | Greece |
| 158 | MW503094.1 | 37°42'43.2"N 22°16'55.2"E |
| 159 | MW502266.1 | 40°57'39.6"N 26°03'57.6"E |
| 160 | MW501547.1 | 40°12'18.0"N 21°03'50.4"E |
| 161 | MW500371.1 | 40°57'39.6"N 26°03'57.6"E |
| 162 | MW500273.1 | 37°42'43.2"N 22°16'55.2"E |
| 163 | MW499843.1 | 40°57'39.6"N 26°03'57.6"E |
| 164 | MW499016.1 | 40°12'18.0"N 21°03'50.4"E |
| 165 | MN182262.1 | 41°18'36.0"N 24°03'00.0"E |
| 166 | MN182255.1 | 40°57'39.6"N 26°03'57.6"E |
| 167 | MN182254.1 | 37°42'43.2"N 22°16'55.2"E |
| 168 | MN182253.1 | 40°57'39.6"N 26°03'57.6"E |
| 169 | MN182251.1 | 40°12'18.0"N 21°03'50.4"E |
| 170 | MN182250.1 | 40°19'12.0"N 21°39'00.0"E |
| 171 | MN182249.1 | 35°18'00.0"N 23°42'00.0"E |
| 172 | MN182248.1 | 35°18'00.0"N 23°42'00.0"E |
| 173 | MN182073.1 | 35°18'00.0"N 23°42'00.0"E |
| 174 | MW503560.1 | 42°04'12.0"N 11°56'24.0"E | Italy |
| 175 | MW502548.1 | 43°51'57.6"N 11°43'48.0"E |
| 176 | MW502323.1 | 42°04'12.0"N 11°56'24.0"E |
| 177 | MW500136.1 | 43°51'57.6"N 11°43'48.0"E |
| 178 | MW499669.1 | 42°04'12.0"N 11°56'24.0"E |
| 179 | MW499237.1 | 43°51'57.6"N 11°43'48.0"E |
| 180 | MW499058.1 | 43°49'58.8"N 11°43'58.8"E |
| 181 | MN145418.1 | 42°04'12.0"N 11°56'24.0"E |
| 182 | MN145219.1 | 43°51'57.6"N 11°43'48.0"E |
| 183 | MN145190.1 | 42°04'12.0"N 11°56'24.0"E |
| 184 | MN144969.1 | 43°51'57.6"N 11°43'48.0"E |
| 185 | MN144699.1 | 42°04'12.0"N 11°56'24.0"E |
| 186 | MN144618.1 | 43°51'57.6"N 11°43'48.0"E |
| 187 | MN144484.1 | 43°49'58.8"N 11°43'58.8"E |
| 188 | MN144128.1 | 43°51'57.6"N 11°43'48.0"E |
| 189 | MN144118.1 | 43°49'58.8"N 11°43'58.8"E |
| 190 | MN144000.1 | 45°39'46.8"N 8°05'24.0"E |
| 191 | MN143992.1 | 45°39'46.8"N 8°05'24.0"E |
| 192 | MN143823.1 | 45°39'46.8"N 8°05'24.0"E |
| 193 | MN143793.1 | 45°39'46.8"N 8°05'24.0"E |
| 194 | MN143758.1 | 40°32'52.8"N 14°13'26.4"E |
| 195 | MN143701.1 | 40°32'52.8"N 14°13'26.4"E |
| 196 | MN143639.1 | 40°32'52.8"N 14°13'26.4"E |
| 197 | MN143541.1 | 40°49'04.8"N 15°05'49.2"E |
| 198 | MN143343.1 | 40°49'04.8"N 15°05'49.2"E |
| 199 | MN143334.1 | 40°49'04.8"N 15°05'49.2"E |
| 200 | MN143279.1 | 40°49'04.8"N 15°05'49.2"E |

| Sl. NO | Accession | GPS | Country |
| --- | --- | --- | --- |
| 201 | MN142967.1 | 40°33'00.0"N 14°14'16.8"E | Italy |
| 202 | MN142959.1 | 40°32'52.8"N 14°13'26.4"E |
| 203 | MN142949.1 | 40°32'52.8"N 14°13'26.4"E |
| 204 | MN142827.1 | 40°32'52.8"N 14°13'26.4"E |
| 205 | MN142677.1 | 40°32'52.8"N 14°13'26.4"E |
| 206 | MN142252.1 | 40°32'52.8"N 14°13'26.4"E |
| 207 | MN142249.1 | 40°32'52.8"N 14°13'26.4"E |
| 208 | MN142137.1 | 40°32'52.8"N 14°13'26.4"E |
| 209 | MN141684.1 | 40°32'52.8"N 14°13'26.4"E |
| 210 | MN141409.1 | 46°48'50.4"N 11°56'45.6"E |
| 211 | MN141163.1 | 40°33'00.0"N 14°14'16.8"E |
| 212 | MN140902.1 | 40°32'52.8"N 14°13'26.4"E |
| 213 | MN140348.1 | 40°32'52.8"N 14°13'26.4"E |
| 214 | MN140173.1 | 40°32'52.8"N 14°13'26.4"E |
| 215 | MN140162.1 | 40°32'52.8"N 14°13'26.4"E |
| 216 | MN139968.1 | 40°32'52.8"N 14°13'26.4"E |
| 217 | MN139868.1 | 40°32'52.8"N 14°13'26.4"E |
| 218 | MN139866.1 | 40°32'52.8"N 14°13'26.4"E |
| 219 | MN139737.1 | 40°32'52.8"N 14°13'26.4"E |
| 220 | MN139727.1 | 46°48'50.4"N 11°56'45.6"E |
| 221 | MN139603.1 | 40°33'00.0"N 14°14'16.8"E |
| 222 | MN139448.1 | 40°32'52.8"N 14°13'26.4"E |
| 223 | MN139436.1 | 40°32'52.8"N 14°13'26.4"E |
| 224 | MN139309.1 | 40°32'52.8"N 14°13'26.4"E |
| 225 | MN139295.1 | 40°32'52.8"N 14°13'26.4"E |
| 226 | MN139282.1 | 40°32'52.8"N 14°13'26.4"E |
| 227 | MN138913.1 | 40°32'52.8"N 14°13'26.4"E |
| 228 | MN138870.1 | 40°32'52.8"N 14°13'26.4"E |
| 229 | MN138792.1 | 40°32'52.8"N 14°13'26.4"E |
| 230 | MN138753.1 | 46°48'50.4"N 11°56'45.6"E |
| 231 | MN138593.1 | 40°32'52.8"N 14°13'26.4"E |
| 232 | MN138559.1 | 40°32'52.8"N 14°13'26.4"E |
| 233 | MN138554.1 | 40°32'52.8"N 14°13'26.4"E |
| 234 | MN138535.1 | 40°32'52.8"N 14°13'26.4"E |
| 235 | MN138523.1 | 40°32'52.8"N 14°13'26.4"E |
| 236 | MN138503.1 | 40°32'52.8"N 14°13'26.4"E |
| 237 | MN182287.1 | 46°48'50.4"N 11°56'45.6"E |
| 238 | MN182286.1 | 40°32'52.8"N 14°13'26.4"E |
| 239 | MN182285.1 | 40°32'52.8"N 14°13'26.4"E |
| 240 | MN182282.1 | 40°32'52.8"N 14°13'26.4"E |
| 241 | MN182283.1 | 40°32'52.8"N 14°13'26.4"E |
| 242 | MN182284.1 | 40°32'52.8"N 14°13'26.4"E |
| 243 | MN182264.1 | 40°32'52.8"N 14°13'26.4"E |
| 244 | MN182245.1 | 46°48'50.4"N 11°56'45.6"E |
| 245 | MN182238.1 | 41°10'08.4"N 9°18'07.2"E |
| 246 | MN182232.1 | 41°10'08.4"N 9°18'07.2"E |
| 247 | MN182219.1 | 41°10'08.4"N 9°18'07.2"E |
| 248 | MH420397.1 | 41°10'08.4"N 9°18'07.2"E |
| 249 | MH420391.1 | 41°10'08.4"N 9°18'07.2"E |
| 250 | MH420352.1 | 41°10'08.4"N 9°18'07.2"E |
| 251 | MH420350.1 | 40°32'52.8"N 14°13'26.4"E |
| 252 | MH420330.1 | 40°32'52.8"N 14°13'26.4"E |
| 253 | MH420286.1 | 40°32'52.8"N 14°13'26.4"E |
| 254 | MH420266.1 | 40°32'52.8"N 14°13'26.4"E |
| 255 | MH420274.1 | 40°32'52.8"N 14°13'26.4"E |
| 256 | MH420210.1 | 46°48'50.4"N 11°56'45.6"E |
| 257 | MH420205.1 | 41°10'08.4"N 9°18'07.2"E |
| 258 | MH420114.1 | 41°10'08.4"N 9°18'07.2"E |
| 259 | MH420121.1 | 41°10'08.4"N 9°18'07.2"E |
| 260 | MH420169.1 | 41°10'08.4"N 9°18'07.2"E |
| 261 | MH420174.1 | 41°10'08.4"N 9°18'07.2"E |
| 262 | MH420095.1 | 41°10'08.4"N 9°18'07.2"E |
| 263 | MH420009.1 | 41°10'08.4"N 9°18'07.2"E |
| 264 | MH419962.1 | 36°47'06.0"N 11°58'51.6"E |
| 265 | MH419953.1 | 36°47'06.0"N 11°58'51.6"E |
| 266 | MH419942.1 | 36°47'06.0"N 11°58'51.6"E |
| 267 | MH419926.1 | 36°47'06.0"N 11°58'51.6"E |
| 268 | MH419871.1 | 36°47'06.0"N 11°58'51.6"E |
| 269 | MH419865.1 | 36°47'06.0"N 11°58'51.6"E |
| 270 | MH419853.1 | 36°47'06.0"N 11°58'51.6"E |
| 271 | MH419839.1 | 36°47'06.0"N 11°58'51.6"E |
| 272 | MH419815.1 | 36°47'06.0"N 11°58'51.6"E |
| 273 | MH419770.1 | 36°47'06.0"N 11°58'51.6"E |
| 274 | MH419742.1 | 36°47'06.0"N 11°58'51.6"E |
| 275 | MH419727.1 | 36°47'06.0"N 11°58'51.6"E |
| 276 | MH419719.1 | 36°47'06.0"N 11°58'51.6"E |
| 277 | MH419654.1 | 36°47'06.0"N 11°58'51.6"E |
| 278 | MH419639.1 | 36°47'06.0"N 11°58'51.6"E |
| 279 | MH419600.1 | 36°47'06.0"N 11°58'51.6"E |
| 280 | MH419574.1 | 37°04'04.8"N 14°21'00.0"E |
| 281 | MH419559.1 | 37°04'04.8"N 14°21'00.0"E |
| 282 | MH419554.1 | 37°04'04.8"N 14°21'00.0"E |
| 283 | MH419529.1 | 37°04'04.8"N 14°21'00.0"E |
| 284 | MH419509.1 | 37°04'04.8"N 14°21'00.0"E |
| 285 | MH419497.1 | 37°04'04.8"N 14°21'00.0"E |
| 286 | MH419491.1 | 37°04'04.8"N 14°21'00.0"E |
| 287 | MH419374.1 | 37°04'04.8"N 14°21'00.0"E |
| 288 | MH419334.1 | 37°04'04.8"N 14°21'00.0"E |
| 289 | MH419298.1 | 37°58'12.0"N 14°46'12.0"E |
| 290 | MH419199.1 | 37°58'12.0"N 14°46'12.0"E |
| 291 | MH419139.1 | 37°58'12.0"N 14°46'12.0"E |
| 292 | MH419073.1 | 37°58'12.0"N 14°46'12.0"E |
| 293 | MH419069.1 | 37°58'12.0"N 14°46'12.0"E |
| 294 | MH419040.1 | 37°58'12.0"N 14°46'12.0"E |
| 295 | MH419031.1 | 37°58'12.0"N 14°46'12.0"E |
| 296 | MH419016.1 | 37°58'12.0"N 14°46'12.0"E |
| 297 | MH419003.1 | 37°58'12.0"N 14°46'12.0"E |
| 298 | MH418966.1 | 37°58'12.0"N 14°46'12.0"E |
| 299 | MH418965.1 | 37°58'12.0"N 14°46'12.0"E |
| 300 | MH418936.1 | 37°58'12.0"N 14°46'12.0"E |

| Sl. NO | Accession | GPS | Country |
| --- | --- | --- | --- |
| 301 | MH418917.1 | 38°43'19.2"N 16°00'46.8"E | Italy |
| 302 | MH418908.1 | 38°43'19.2"N 16°00'46.8"E |
| 303 | MH418914.1 | 38°43'19.2"N 16°00'46.8"E |
| 304 | MH418889.1 | 38°43'19.2"N 16°00'46.8"E |
| 305 | MH418861.1 | 38°43'19.2"N 16°00'46.8"E |
| 306 | MH418860.1 | 38°43'19.2"N 16°00'46.8"E |
| 307 | MH418854.1 | 38°43'19.2"N 16°00'46.8"E |
| 308 | MH418842.1 | 38°43'19.2"N 16°00'46.8"E |
| 309 | MH418833.1 | 38°43'19.2"N 16°00'46.8"E |
| 310 | MH418828.1 | 38°43'19.2"N 16°00'46.8"E |
| 311 | MH418752.1 | 38°43'19.2"N 16°00'46.8"E |
| 312 | MH418751.1 | 38°43'19.2"N 16°00'46.8"E |
| 313 | MH418731.1 | 38°43'19.2"N 16°00'46.8"E |
| 314 | MH418718.1 | 38°43'19.2"N 16°00'46.8"E |
| 315 | MH418677.1 | 39°52'01.2"N 9°16'01.2"E |
| 316 | MH418663.1 | 39°52'01.2"N 9°16'01.2"E |
| 317 | MH418636.1 | 39°52'01.2"N 9°16'01.2"E |
| 318 | MH418576.1 | 39°52'01.2"N 9°16'01.2"E |
| 319 | MH418624.1 | 39°52'01.2"N 9°16'01.2"E |
| 320 | MH418575.1 | 39°52'01.2"N 9°16'01.2"E |
| 321 | MH418520.1 | 39°52'01.2"N 9°16'01.2"E |
| 322 | MH418515.1 | 39°52'01.2"N 9°16'01.2"E |
| 323 | MH418495.1 | 39°52'01.2"N 9°16'01.2"E |
| 324 | MN182218.1 | 36°02'24.0"N 14°16'12.0"E | Malta |
| 325 | MN182217.1 | 36°02'24.0"N 14°16'12.0"E |
| 326 | MN182216.1 | 35°54'00.0"N 14°24'36.0"E |
| 327 | MN182214.1 | 35°54'00.0"N 14°24'36.0"E |
| 328 | MH420135.1 | 36°02'31.2"N 14°16'26.4"E |
| 329 | MH419828.1 | 36°02'31.2"N 14°16'26.4"E |
| 330 | MH420314.1 | 36°02'31.2"N 14°16'26.4"E |
| 331 | MH419630.1 | 36°02'31.2"N 14°16'26.4"E |
| 332 | MH419690.1 | 36°02'31.2"N 14°16'26.4"E |
| 333 | MH419360.1 | 36°02'31.2"N 14°16'26.4"E |
| 334 | MH419099.1 | 36°02'31.2"N 14°16'26.4"E |
| 335 | MW501927.1 | 52°49'08.4"N 14°14'04.6"E | Poland |
| 336 | MW500203.1 | 52°49'08.4"N 14°14'04.6"E |
| 337 | MN182231.1 | 52°52'12.0"N 14°12'00.0"E |
| 338 | MN182230.1 | 52°18'00.0"N 16°53'24.0"E |
| 339 | MN182229.1 | 52°18'00.0"N 16°53'24.0"E |
| 340 | MN182228.1 | 52°18'00.0"N 16°53'24.0"E |
| 341 | MN182227.1 | 52°18'00.0"N 16°53'24.0"E |
| 342 | MN182226.1 | 52°18'00.0"N 16°53'24.0"E |
| 343 | MN182225.1 | 49°36'36.0"N 19°30'00.0"E |
| 344 | MN182224.1 | 49°36'36.0"N 19°30'00.0"E |
| 345 | MN182223.1 | 49°36'36.0"N 19°30'00.0"E |
| 346 | MN182222.1 | 49°36'36.0"N 19°30'00.0"E |
| 347 | MN182221.1 | 49°36'36.0"N 19°30'00.0"E |
| 348 | MN182220.1 | 49°36'36.0"N 19°30'00.0"E |
| 349 | MN182252.1 | 44°28'12.0"N 28°29'24.0"E | Romania |
| 350 | MN182213.1 | 47°45'36.0"N 23°36'36.0"E |
| 351 | MN182205.1 | 47°45'36.0"N 23°36'36.0"E |
| 352 | MN182204.1 | 47°45'36.0"N 23°36'36.0"E |
| 353 | MN182203.1 | 47°45'36.0"N 23°36'36.0"E |
| 354 | MN182199.1 | 47°45'36.0"N 23°36'36.0"E |
| 355 | MN182200.1 | 47°10'48.0"N 27°28'48.0"E |
| 356 | MN182201.1 | 45°10'48.0"N 22°18'00.0"E |
| 357 | MN182202.1 | 44°03'36.0"N 27°39'00.0"E |
| 358 | HQ004961.1 | 45°01'48.0"N 28°31'12.0"E |
| 359 | HQ004962.1 | 45°01'48.0"N 28°31'12.0"E |
| 360 | HQ004960.1 | 45°01'48.0"N 28°31'12.0"E |
| 361 | HQ004959.1 | 45°01'48.0"N 28°31'12.0"E |
| 362 | HQ004958.1 | 45°01'48.0"N 28°31'12.0"E |
| 363 | HQ004957.1 | 45°07'48.0"N 26°37'48.0"E |
| 364 | HQ004956.1 | 45°07'48.0"N 26°37'48.0"E |
| 365 | HQ004953.1 | 45°07'48.0"N 26°37'48.0"E |
| 366 | HQ004954.1 | 45°07'48.0"N 26°37'48.0"E |
| 367 | HQ004955.1 | 45°07'48.0"N 26°37'48.0"E |
| 368 | MW502742.1 | 42°47'24.5"N 0°28'51.5"W | Spain |
| 369 | MW502012.1 | 42°47'24.5"N 0°28'51.5"W |
| 370 | GU675871.1 | 28°22'00.1"N 16°43'19.9"W |
| 371 | MN141453.1 | 42°25'01.2"N 5°18'18.0"W |
| 372 | MN144940.1 | 42°47'24.5"N 0°28'51.5"W |
| 373 | MN144664.1 | 42°47'24.5"N 0°28'51.5"W |
| 374 | MN143579.1 | 28°22'00.1"N 16°43'19.9"W |
| 375 | MN141439.1 | 42°25'01.2"N 5°18'18.0"W |
| 376 | MN141230.1 | 42°47'24.5"N 0°28'51.5"W |
| 377 | MN141198.1 | 42°47'24.5"N 0°28'51.5"W |
| 378 | MN139651.1 | 28°22'00.1"N 16°43'19.9"W |
| 379 | MN139365.1 | 42°25'01.2"N 5°18'18.0"W |
| 380 | MN139182.1 | 42°47'24.5"N 0°28'51.5"W |
| 381 | MN138573.1 | 42°47'24.5"N 0°28'51.5"W |
| 382 | MN138567.1 | 28°22'00.1"N 16°43'19.9"W |
| 383 | MN182244.1 | 42°25'01.2"N 5°18'18.0"W |
| 384 | MN182243.1 | 42°47'24.5"N 0°28'51.5"W |
| 385 | MN182237.1 | 42°47'24.5"N 0°28'51.5"W |
| 386 | MN182236.1 | 28°22'00.1"N 16°43'19.9"W |
| 387 | MN182234.1 | 42°25'01.2"N 5°18'18.0"W |
| 388 | MN182235.1 | 42°47'24.5"N 0°28'51.5"W |
| 389 | MN182233.1 | 42°47'24.5"N 0°28'51.5"W |
| 390 | MN182215.1 | 28°22'00.1"N 16°43'19.9"W |
| 391 | MN182207.1 | 42°25'01.2"N 5°18'18.0"W |
| 392 | MN182206.1 | 42°47'24.5"N 0°28'51.5"W |
| 393 | MN182191.1 | 42°47'24.5"N 0°28'51.5"W |
| 394 | MN182190.1 | 28°22'00.1"N 16°43'19.9"W |
| 395 | MN182189.1 | 42°25'01.2"N 5°18'18.0"W |
| 396 | MN182185.1 | 42°47'24.5"N 0°28'51.5"W |
| 397 | MN182187.1 | 42°47'24.5"N 0°28'51.5"W |
| 398 | MN182186.1 | 28°22'00.1"N 16°43'19.9"W |
| 399 | MN182188.1 | 42°25'01.2"N 5°18'18.0"W |
| 400 | MN181891.1 | 42°00'36.0"N 2°28'12.0"E |

| 401 | MN181890.1 | 41°42'00.0"N 2°25'48.0"E | Spain |
| --- | --- | --- | --- |
| 402 | MN181889.1 | 41°42'00.0"N 2°25'48.0"E |
| 403 | MN181888.1 | 40°54'00.0"N 4°01'12.0"W |
| 404 | MN181887.1 | 40°54'00.0"N 4°01'12.0"W |
| 405 | GU669670.1 | 41°46'26.0"N 2°22'47.0"E |
| 406 | GU669669.1 | 41°47'29.8"N 2°20'05.9"E |
| 407 | GU676736.1 | 41°46'26.0"N 2°22'47.0"E |
| 408 | GU676848.1 | 41°47'29.8"N 2°20'05.9"E |
| 409 | GU669615.1 | 41°46'26.0"N 2°22'47.0"E |
| 410 | GU669619.1 | 41°47'29.8"N 2°20'05.9"E |
| 411 | GU669620.1 | 41°46'26.0"N 2°22'47.0"E |
| 412 | GU676602.1 | 41°47'29.8"N 2°20'05.9"E |
| 413 | GU676659.1 | 41°46'26.0"N 2°22'47.0"E |
| 414 | GU676719.1 | 41°47'29.8"N 2°20'05.9"E |
| 415 | GU676534.1 | 41°46'26.0"N 2°22'47.0"E |
| 416 | GU676461.1 | 41°47'29.8"N 2°20'05.9"E |
| 417 | GU676400.1 | 40°25'43.7"N 0°16'05.4"W |
| 418 | GU676446.1 | 40°25'43.7"N 0°16'05.4"W |
| 419 | GU676452.1 | 40°25'43.7"N 0°16'05.4"W |
| 420 | GU675894.1 | 40°25'43.7"N 0°16'05.4"W |
| 421 | GU675995.1 | 40°25'43.7"N 0°16'05.4"W |
| 422 | GU675996.1 | 40°25'43.7"N 0°16'05.4"W |
| 423 | GU675998.1 | 39°37'59.5"N 2°39'03.2"E |
| 424 | GU676386.1 | 39°37'59.5"N 2°39'03.2"E |
| 425 | GU675686.1 | 39°37'59.5"N 2°39'03.2"E |
| 426 | GU675841.1 | 39°37'59.5"N 2°39'03.2"E |
| 427 | GU675844.1 | 39°37'59.5"N 2°39'03.2"E |
| 428 | MN138718.1 | 38°52'19.2"N 1°23'52.8"E |
| 429 | JN827888.1 | 41°44'12.1"N 2°19'42.0"E |
| 430 | KP871138.1 | 41°18'00.0"N 4°54'36.0"W |
| 431 | KP870909.1 | 41°18'00.0"N 4°54'36.0" |
| 432 | KP870897.1 | 41°18'00.0"N 4°54'36.0" |
| 433 | KP870788.1 | 41°18'00.0"N 4°54'36.0" |
| 434 | KP870597.1 | 41°18'00.0"N 4°54'36.0" |
| 435 | KP870579.1 | 39°53'52.8"N 0°41'02.4"E |
| 436 | KP870279.1 | 39°53'52.8"N 0°41'02.4"E |
| 437 | KP870276.1 | 39°53'52.8"N 0°41'02.4"E |
| 438 | MW502130.1 | 51°44'20.0"N 0°47'56.0"W | United Kingdom |
| 439 | MW502038.1 | 51°44'45.6"N 1°12'32.4"W |
| 440 | MW501659.1 | 51°44'45.6"N 1°12'32.4"W |
| 441 | MW501412.1 | 51°44'45.6"N 1°12'32.4"W |
| 442 | MW501185.1 | 51°44'45.6"N 1°12'32.4"W |
| 443 | MW500842.1 | 51°44'45.6"N 1°12'32.4"W |
| 444 | MW499555.1 | 51°44'45.6"N 1°12'32.4"W |
| 445 | MW499236.1 | 51°44'45.6"N 1°12'32.4"W |
| 446 | MN143974.1 | 54°41'24.0"N 1°29'27.6"W |
| 447 | MN141416.1 | 54°41'24.0"N 1°29'27.6"W |
| 448 | MN182295.1 | 54°41'24.0"N 1°29'27.6"W |
| 449 | MN182281.1 | 54°41'24.0"N 1°29'27.6"W |
| 450 | MN182280.1 | 54°41'24.0"N 1°29'27.6"W |
| 451 | MN182279.1 | 54°41'24.0"N 1°29'27.6"W |
| 452 | MN182278.1 | 52°00'36.0"N 0°15'00.0"E |
| 453 | MN182277.1 | 52°00'36.0"N 0°15'00.0"E |
| 454 | MN182276.1 | 52°00'36.0"N 0°15'00.0"E |
| 455 | MN182275.1 | 52°00'36.0"N 0°15'00.0"E |
| 456 | MN182274.1 | 52°00'36.0"N 0°15'00.0"E |
| 457 | MN182273.1 | 52°00'36.0"N 0°15'00.0"E |
| 458 | MN182272.1 | 52°00'36.0"N 0°15'00.0"E |
| 459 | MN182267.1 | 52°00'36.0"N 0°15'00.0"E |
| 460 | MN182268.1 | 52°00'36.0"N 0°15'00.0"E |
| 461 | MN182269.1 | 52°00'36.0"N 0°15'00.0"E |
| 462 | MN182270.1 | 52°00'36.0"N 0°15'00.0"E |
| 463 | MN182271.1 | 52°00'36.0"N 0°15'00.0"E |
| 464 | MN182247.1 | 51°57'00.0"N 0°26'24.0"E |
| 465 | MN182246.1 | 51°57'00.0"N 0°26'24.0"E |
| 466 | MN182194.1 | 51°57'00.0"N 0°26'24.0"E |
| 467 | MN182193.1 | 51°57'00.0"N 0°26'24.0"E |
| 468 | MN181901.1 | 51°57'00.0"N 0°26'24.0"E |
| 469 | MN181900.1 | 51°57'00.0"N 0°26'24.0"E |
| 470 | MN181899.1 | 51°57'00.0"N 0°26'24.0"E |

| Sl. No | Accession | GPS | Country |
| --- | --- | --- | --- |
| 1 | GU673011.1 | 58°44'02.4"N 94°06'43.2"W | Canada |
| 2 | GU673008.1 | 58°44'24.0"N 93°49'12.0"W |
| 3 | GU673060.1 | 58°44'24.0"N 93°49'12.0"W |
| 4 | GU672853.1 | 58°44'24.0"N 93°49'12.0"W |
| 5 | MN182325.1 | 43°00'00.0"N 81°17'24.0"W |
| 6 | MN182010.1 | 43°30'00.0"N 80°22'48.0"W |
| 7 | MN181945.1 | 43°30'00.0"N 80°22'48.0"W |
| 8 | MN181944.1 | 43°00'00.0"N 81°17'24.0"W |
| 9 | MN181942.1 | 43°30'00.0"N 80°22'48.0"W |
| 10 | MN181941.1 | 43°30'00.0"N 80°22'48.0"W |
| 11 | MN181940.1 | 43°00'00.0"N 81°17'24.0"W |
| 12 | MN181939.1 | 43°30'00.0"N 80°22'48.0"W |
| 13 | MN181938.1 | 43°30'00.0"N 80°22'48.0"W |
| 14 | MN181914.1 | 43°00'00.0"N 81°17'24.0"W |
| 15 | MN181762.1 | 43°30'00.0"N 80°22'48.0"W |
| 16 | MN181761.1 | 43°30'00.0"N 80°22'48.0"W |
| 17 | MN181759.1 | 43°00'00.0"N 81°17'24.0"W |
| 18 | MN181760.1 | 43°30'00.0"N 80°22'48.0"W |
| 19 | MN181758.1 | 43°30'00.0"N 80°22'48.0"W |
| 20 | MN181757.1 | 43°00'00.0"N 81°17'24.0"W |
| 21 | MN181756.1 | 43°30'00.0"N 80°22'48.0"W |
| 22 | MN181755.1 | 43°30'00.0"N 80°22'48.0"W |
| 23 | MN181673.1 | 43°00'00.0"N 81°17'24.0"W |
| 24 | MN181671.1 | 43°30'00.0"N 80°22'48.0"W |
| 25 | MN181647.1 | 43°30'00.0"N 80°22'48.0"W |
| 26 | MN181645.1 | 43°30'00.0"N 80°22'48.0"W |
| 27 | KM554514.1 | 45°11'24.0"N 81°34'48.0"W |
| 28 | KM549875.1 | 43°30'00.0"N 80°22'48.0"W |
| 29 | KM547147.1 | 45°11'24.0"N 81°34'48.0"W |
| 30 | KM545769.1 | 41°55'48.0"N 82°30'36.0"W |
| 31 | KM545461.1 | 41°55'48.0"N 82°30'36.0"W |
| 32 | KM540572.1 | 41°55'48.0"N 82°30'36.0"W |
| 33 | KM541554.1 | 41°55'48.0"N 82°30'36.0"W |
| 34 | KM544400.1 | 50°52'22.8"N 100°03'00.0"W |
| 35 | KM545294.1 | 50°52'22.8"N 100°03'00.0"W |
| 36 | KM545315.1 | 50°52'22.8"N 100°03'00.0"W |
| 37 | KM540520.1 | 50°52'22.8"N 100°03'00.0"W |
| 38 | HM415246.1 | 53°41'13.2"N 112°48'46.8"W |
| 39 | JF841240.1 | 53°41'13.2"N 112°48'46.8"W |
| 40 | JF841241.1 | 53°41'13.2"N 112°48'46.8"W |
| 41 | KJ165179.1 | 41°56'20.4"N 82°30'57.6"W |
| 42 | GU092010.1 | 43°32'13.2"N 80°08'07.1"W |
| 43 | HM415245.1 | 43°32'13.2"N 80°08'07.1"W |
| 44 | GU092011.1 | 43°32'13.2"N 80°08'07.1"W |
| 45 | KT142082.1 | 51°09'32.4"N 116°47'45.6"W |
| 46 | KT140090.1 | 51°09'32.4"N 116°47'45.6"W |
| 47 | KT138981.1 | 51°09'32.4"N 116°47'45.6"W |
| 48 | KT135786.1 | 51°09'32.4"N 116°47'45.6"W |
| 49 | KT127776.1 | 51°09'32.4"N 116°47'45.6"W |
| 50 | KX281214.1 | 50°18'20.9"N 111°47'16.2"W |
| 51 | HQ583584.1 | 38°42'24.8"N 104°50'09.6"W | USA |
| 52 | HQ583583.1 | 38°42'24.8"N 104°50'09.6"W |
| 53 | HQ583582.1 | 38°42'24.8"N 104°50'09.6"W |
| 54 | HQ583581.1 | 38°42'24.8"N 104°50'09.6"W |
| 55 | HQ583580.1 | 38°42'24.8"N 104°50'09.6"W |
| 56 | MN182331.1 | 38°55'12.0"N 77°24'00.0"W |
| 57 | MN182329.1 | 38°55'12.0"N 77°24'00.0"W |
| 58 | MN182330.1 | 38°55'12.0"N 77°24'00.0"W |
| 59 | MN182328.1 | 38°55'12.0"N 77°24'00.0"W |
| 60 | MN182327.1 | 38°55'12.0"N 77°24'00.0"W |
| 61 | MN182326.1 | 38°42'24.8"N 104°50'09.6"W |
| 62 | MN182324.1 | 38°42'24.8"N 104°50'09.6"W |
| 63 | MN182323.1 | 38°42'24.8"N 104°50'09.6"W |
| 64 | MN182322.1 | 38°42'24.8"N 104°50'09.6"W |
| 65 | MN182321.1 | 38°42'24.8"N 104°50'09.6"W |
| 66 | MN182320.1 | 38°55'12.0"N 77°24'00.0"W |
| 67 | MN182241.1 | 38°55'12.0"N 77°24'00.0"W |
| 68 | MN182166.1 | 38°55'12.0"N 77°24'00.0"W |
| 69 | MN182165.1 | 38°55'12.0"N 77°24'00.0"W |
| 70 | MN182164.1 | 38°55'12.0"N 77°24'00.0"W |
| 71 | MN182163.1 | 38°42'24.8"N 104°50'09.6"W |
| 72 | MN182162.1 | 38°42'24.8"N 104°50'09.6"W |
| 73 | MN182161.1 | 38°42'24.8"N 104°50'09.6"W |
| 74 | MN182160.1 | 38°42'24.8"N 104°50'09.6"W |
| 75 | MN182159.1 | 38°42'24.8"N 104°50'09.6"W |
| 76 | MN182158.1 | 38°55'12.0"N 77°24'00.0"W |
| 77 | MN182157.1 | 38°55'12.0"N 77°24'00.0"W |
| 78 | MN182156.1 | 38°55'12.0"N 77°24'00.0"W |
| 79 | MN182155.1 | 38°55'12.0"N 77°24'00.0"W |
| 80 | MN182154.1 | 38°55'12.0"N 77°24'00.0"W |
| 81 | MN182153.1 | 39°28'48.0"N 118°40'12.0"W |
| 82 | MN182152.1 | 39°28'48.0"N 118°40'12.0"W |
| 83 | MN182151.1 | 39°41'24.0"N 119°49'48.0"W |
| 84 | MN182150.1 | 39°41'24.0"N 119°49'48.0"W |
| 85 | MN182149.1 | 39°41'24.0"N 119°49'48.0"W |
| 86 | MN182148.1 | 39°41'24.0"N 119°49'48.0"W |
| 87 | MN182147.1 | 39°41'24.0"N 119°49'48.0"W |
| 88 | MN182146.1 | 39°41'24.0"N 119°49'48.0"W |
| 89 | MN182145.1 | 39°41'24.0"N 119°49'48.0"W |
| 90 | MN182144.1 | 39°41'24.0"N 119°49'48.0"W |
| 91 | MN182143.1 | 38°10'48.0"N 122°01'48.0"W |
| 92 | MN182142.1 | 38°10'48.0"N 122°01'48.0"W |
| 93 | MN182141.1 | 38°10'48.0"N 122°01'48.0"W |
| 94 | MN182140.1 | 38°10'48.0"N 122°01'48.0"W |
| 95 | MN182139.1 | 38°10'48.0"N 122°01'48.0"W |
| 96 | MN182138.1 | 21°24'36.0"N 157°48'00.0"W |
| 97 | MN182137.1 | 21°24'36.0"N 157°48'00.0"W |
| 98 | MN182136.1 | 21°24'36.0"N 157°48'00.0"W |
| 99 | MN182135.1 | 21°24'36.0"N 157°48'00.0"W |
| 100 | MN182134.1 | 21°24'36.0"N 157°48'00.0"W |

| Sl. No | Accession | GPS | Country |
| --- | --- | --- | --- |
| 101 | MN182133.1 | 21°24'36.0"N 157°48'00.0"W | USA |
| 102 | MN182132.1 | 21°24'36.0"N 157°48'00.0"W |
| 103 | MN182131.1 | 21°24'36.0"N 157°48'00.0"W |
| 104 | MN182130.1 | 21°24'36.0"N 157°48'00.0"W |
| 105 | MN182129.1 | 37°42'36.0"N 122°29'24.0"W |
| 106 | MN182128.1 | 37°42'36.0"N 122°29'24.0"W |
| 107 | MN182127.1 | 37°42'36.0"N 122°29'24.0"W |
| 108 | MN182126.1 | 37°42'36.0"N 122°29'24.0"W |
| 109 | MN182125.1 | 37°42'36.0"N 122°29'24.0"W |
| 110 | MN182124.1 | 37°42'00.0"N 122°28'12.0"W |
| 111 | MN182123.1 | 37°42'36.0"N 122°29'24.0"W |
| 112 | MN182122.1 | 37°42'36.0"N 122°29'24.0"W |
| 113 | MN182121.1 | 37°42'36.0"N 122°29'24.0"W |
| 114 | MN182120.1 | 37°42'00.0"N 122°28'12.0"W |
| 115 | MN182047.1 | 44°14'24.0"N 69°21'36.0"W |
| 116 | MN182046.1 | 44°14'24.0"N 69°21'36.0"W |
| 117 | MN182045.1 | 44°14'24.0"N 69°21'36.0"W |
| 118 | MN182041.1 | 44°14'24.0"N 69°21'36.0"W |
| 119 | MN182040.1 | 44°14'24.0"N 69°21'36.0"W |
| 120 | MN182033.1 | 45°19'12.0"N 92°31'48.0"W |
| 121 | MN182023.1 | 45°19'12.0"N 92°31'48.0"W |
| 122 | MN182022.1 | 32°40'48.0"N 117°10'48.0"W |
| 123 | MN182021.1 | 32°40'48.0"N 117°10'48.0"W |
| 124 | MN182015.1 | 32°40'48.0"N 117°10'48.0"W |
| 125 | MN182013.1 | 32°40'48.0"N 117°10'48.0"W |
| 126 | MN182012.1 | 43°07'12.0"N 73°42'00.0"W |
| 127 | MN182011.1 | 43°07'12.0"N 73°42'00.0"W |
| 128 | MN182009.1 | 43°07'12.0"N 73°42'00.0"W |
| 129 | MN182008.1 | 43°07'12.0"N 73°42'00.0"W |
| 130 | MN182007.1 | 43°07'12.0"N 73°42'00.0"W |
| 131 | MN182006.1 | 40°28'48.0"N 96°42'36.0"W |
| 132 | MN182005.1 | 40°23'24.0"N 89°55'12.0"W |
| 133 | MN182004.1 | 40°23'24.0"N 89°55'12.0"W |
| 134 | MN182003.1 | 40°23'24.0"N 89°55'12.0"W |
| 135 | MN182002.1 | 45°06'36.0"N 64°23'24.0"W |
| 136 | MN181996.1 | 45°06'36.0"N 64°23'24.0"W |
| 137 | MN181995.1 | 45°06'36.0"N 64°23'24.0"W |
| 138 | MN181994.1 | 45°06'36.0"N 64°23'24.0"W |
| 139 | MN181986.1 | 45°06'36.0"N 64°23'24.0"W |
| 140 | MN181985.1 | 45°31'48.0"N 84°28'12.0"W |
| 141 | MN181984.1 | 45°06'36.0"N 64°23'24.0"W |
| 142 | MN181983.1 | 45°06'36.0"N 64°23'24.0"W |
| 143 | MN181982.1 | 45°06'36.0"N 64°23'24.0"W |
| 144 | MN181981.1 | 45°06'36.0"N 64°23'24.0"W |
| 145 | MN181980.1 | 45°06'36.0"N 64°23'24.0"W |
| 146 | MN181979.1 | 45°06'36.0"N 64°23'24.0"W |
| 147 | MN181978.1 | 45°06'36.0"N 64°23'24.0"W |
| 148 | MN181977.1 | 45°06'36.0"N 64°23'24.0"W |
| 149 | MN181976.1 | 45°06'36.0"N 64°23'24.0"W |
| 150 | MN181974.1 | 45°06'36.0"N 64°23'24.0"W |
| 151 | MN181975.1 | 45°31'48.0"N 84°28'12.0"W |
| 152 | MN181973.1 | 40°23'24.0"N 89°55'12.0"W |
| 153 | MN181972.1 | 40°23'24.0"N 89°55'12.0"W |
| 154 | MN181971.1 | 40°23'24.0"N 89°55'12.0"W |
| 155 | MN181946.1 | 39°02'24.0"N 108°31'48.0"W |
| 156 | MN181943.1 | 38°52'48.0"N 95°15'00.0"W |
| 157 | MN181937.1 | 38°52'48.0"N 95°15'00.0"W |
| 158 | MN181936.1 | 38°52'48.0"N 95°15'00.0"W |
| 159 | MN181935.1 | 38°52'48.0"N 95°15'00.0"W |
| 160 | MN181934.1 | 38°52'48.0"N 95°15'00.0"W |
| 161 | MN181933.1 | 38°52'48.0"N 95°15'00.0"W |
| 162 | MN181932.1 | 39°31'12.0"N 118°31'48.0"W |
| 163 | MN181931.1 | 39°31'12.0"N 118°31'48.0"W |
| 164 | MN181930.1 | 39°31'12.0"N 118°31'48.0"W |
| 165 | MN181929.1 | 39°31'12.0"N 118°31'48.0"W |
| 166 | MN181928.1 | 39°31'12.0"N 118°31'48.0"W |
| 167 | MN181927.1 | 39°31'12.0"N 118°31'48.0"W |
| 168 | MN181926.1 | 39°31'12.0"N 118°31'48.0"W |
| 169 | MN181925.1 | 39°31'12.0"N 118°31'48.0"W |
| 170 | MN181924.1 | 39°31'12.0"N 118°31'48.0"W |
| 171 | MN181923.1 | 39°31'12.0"N 118°31'48.0"W |
| 172 | MN181922.1 | 39°31'12.0"N 118°31'48.0"W |
| 173 | MN181921.1 | 39°31'12.0"N 118°31'48.0"W |
| 174 | MN181920.1 | 39°31'12.0"N 118°31'48.0"W |
| 175 | MN181919.1 | 39°31'12.0"N 118°31'48.0"W |
| 176 | MN181918.1 | 39°31'12.0"N 118°31'48.0"W |
| 177 | MN181917.1 | 39°31'12.0"N 118°31'48.0"W |
| 178 | MN181916.1 | 39°31'12.0"N 118°31'48.0"W |
| 179 | MN181915.1 | 39°31'12.0"N 118°31'48.0"W |
| 180 | MN181913.1 | 39°31'12.0"N 118°31'48.0"W |
| 181 | MN181912.1 | 39°31'12.0"N 118°31'48.0"W |
| 182 | MN181911.1 | 39°31'12.0"N 118°31'48.0"W |
| 183 | MN181910.1 | 39°31'12.0"N 118°31'48.0"W |
| 184 | MN181909.1 | 38°17'24.0"N 85°37'48.0"W |
| 185 | MN181908.1 | 38°17'24.0"N 85°37'48.0"W |
| 186 | MN181907.1 | 38°17'24.0"N 85°37'48.0"W |
| 187 | MN181906.1 | 38°17'24.0"N 85°37'48.0"W |
| 188 | MN181903.1 | 38°17'24.0"N 85°37'48.0"W |
| 189 | MN181904.1 | 38°17'24.0"N 85°37'48.0"W |
| 190 | MN181905.1 | 38°17'24.0"N 85°37'48.0"W |
| 191 | MN181897.1 | 38°17'24.0"N 85°37'48.0"W |
| 192 | MN181893.1 | 38°17'24.0"N 85°37'48.0"W |
| 193 | MN181892.1 | 38°17'24.0"N 85°37'48.0"W |
| 194 | MN181883.1 | 38°17'24.0"N 85°37'48.0"W |
| 195 | MN181882.1 | 38°17'24.0"N 85°37'48.0"W |
| 196 | MN181881.1 | 38°17'24.0"N 85°37'48.0"W |
| 197 | MN181880.1 | 38°17'24.0"N 85°37'48.0"W |
| 198 | MN181879.1 | 38°17'24.0"N 85°37'48.0"W |
| 199 | MN181878.1 | 38°17'24.0"N 85°37'48.0"W |
| 200 | MN181877.1 | 38°17'24.0"N 85°37'48.0"W |

| Sl. No | Accession | GPS | Country |
| --- | --- | --- | --- |
| 201 | MN181876.1 | 42°03'36.0"N 87°50'24.0"W | USA |
| 202 | MN181792.1 | 42°03'36.0"N 87°50'24.0"W |
| 203 | MN181791.1 | 42°03'36.0"N 87°50'24.0"W |
| 204 | MN181790.1 | 42°03'36.0"N 87°50'24.0"W |
| 205 | MN181789.1 | 42°03'36.0"N 87°50'24.0"W |
| 206 | MN181788.1 | 42°03'36.0"N 87°50'24.0"W |
| 207 | MN181787.1 | 42°03'36.0"N 87°50'24.0"W |
| 208 | MN181786.1 | 42°03'36.0"N 87°50'24.0"W |
| 209 | MN181785.1 | 42°03'36.0"N 87°50'24.0"W |
| 210 | MN181784.1 | 36°13'48.0"N 79°10'48.0"W |
| 211 | MN181783.1 | 36°13'48.0"N 79°10'48.0"W |
| 212 | MN181782.1 | 36°13'48.0"N 79°10'48.0"W |
| 213 | MN181781.1 | 36°13'48.0"N 79°10'48.0"W |
| 214 | MN181780.1 | 36°13'48.0"N 79°10'48.0"W |
| 215 | MN181779.1 | 36°13'48.0"N 79°10'48.0"W |
| 216 | MN181778.1 | 36°13'48.0"N 79°10'48.0"W |
| 217 | MN181777.1 | 36°13'48.0"N 79°10'48.0"W |
| 218 | MN181776.1 | 36°13'48.0"N 79°10'48.0"W |
| 219 | MN181775.1 | 39°09'36.0"N 108°45'00.0"W |
| 220 | MN181774.1 | 39°09'36.0"N 108°45'00.0"W |
| 221 | MN181773.1 | 39°09'36.0"N 108°45'00.0"W |
| 222 | MN181772.1 | 39°09'36.0"N 108°45'00.0"W |
| 223 | MN181771.1 | 39°09'36.0"N 108°45'00.0"W |
| 224 | MN181770.1 | 39°09'36.0"N 108°45'00.0"W |
| 225 | MN181769.1 | 39°09'36.0"N 108°45'00.0"W |
| 226 | MN181768.1 | 39°09'36.0"N 108°45'00.0"W |
| 227 | MN181767.1 | 39°09'36.0"N 108°45'00.0"W |
| 228 | MN181766.1 | 39°09'36.0"N 108°45'00.0"W |
| 229 | MN181763.1 | 39°09'36.0"N 108°45'00.0"W |
| 230 | MN181764.1 | 39°09'36.0"N 108°45'00.0"W |
| 231 | MN181765.1 | 43°07'12.0"N 73°42'00.0"W |
| 232 | MN181754.1 | 43°07'12.0"N 73°42'00.0"W |
| 233 | MN181753.1 | 43°07'12.0"N 73°42'00.0"W |
| 234 | MN181752.1 | 43°07'12.0"N 73°42'00.0"W |
| 235 | MN181751.1 | 43°07'12.0"N 73°42'00.0"W |
| 236 | MN181746.1 | 43°07'12.0"N 73°42'00.0"W |
| 237 | MN181747.1 | 43°07'12.0"N 73°42'00.0"W |
| 238 | MN181748.1 | 43°07'12.0"N 73°42'00.0"W |
| 239 | MN181749.1 | 43°07'12.0"N 73°42'00.0"W |
| 240 | MN181750.1 | 43°07'12.0"N 73°42'00.0"W |
| 241 | MN181745.1 | 43°07'12.0"N 73°42'00.0"W |
| 242 | MN181744.1 | 35°37'48.0"N 86°15'00.0"W |
| 243 | MN181743.1 | 35°37'48.0"N 86°15'00.0"W |
| 244 | MN181742.1 | 35°37'48.0"N 86°15'00.0"W |
| 245 | MN181741.1 | 35°37'48.0"N 86°15'00.0"W |
| 246 | MN181736.1 | 35°37'48.0"N 86°15'00.0"W |
| 247 | MN181737.1 | 35°37'48.0"N 86°15'00.0"W |
| 248 | MN181738.1 | 35°37'48.0"N 86°15'00.0"W |
| 249 | MN181739.1 | 35°37'48.0"N 86°15'00.0"W |
| 250 | MN181740.1 | 35°37'48.0"N 86°15'00.0"W |
| 251 | MN181735.1 | 43°26'24.0"N 92°23'24.0"W |
| 252 | MN181734.1 | 43°26'24.0"N 92°23'24.0"W |
| 253 | MN181733.1 | 43°26'24.0"N 92°23'24.0"W |
| 254 | MN181732.1 | 43°26'24.0"N 92°23'24.0"W |
| 255 | MN181731.1 | 43°26'24.0"N 92°23'24.0"W |
| 256 | MN181726.1 | 43°26'24.0"N 92°23'24.0"W |
| 257 | MN181727.1 | 43°26'24.0"N 92°23'24.0"W |
| 258 | MN181729.1 | 43°26'24.0"N 92°23'24.0"W |
| 259 | MN181728.1 | 43°26'24.0"N 92°23'24.0"W |
| 260 | MN181730.1 | 43°26'24.0"N 92°23'24.0"W |
| 261 | MN181723.1 | 43°26'24.0"N 92°23'24.0"W |
| 262 | MN181724.1 | 43°26'24.0"N 92°23'24.0"W |
| 263 | MN181725.1 | 45°27'00.0"N 99°20'24.0"W |
| 264 | MN181722.1 | 45°27'00.0"N 99°20'24.0"W |
| 265 | MN181721.1 | 45°27'00.0"N 99°20'24.0"W |
| 266 | MN181720.1 | 45°27'00.0"N 99°20'24.0"W |
| 267 | MN181719.1 | 45°27'00.0"N 99°20'24.0"W |
| 268 | MN181718.1 | 45°27'00.0"N 99°20'24.0"W |
| 269 | MN181717.1 | 45°27'00.0"N 99°20'24.0"W |
| 270 | MN181716.1 | 45°27'00.0"N 99°20'24.0"W |
| 271 | MN181715.1 | 45°27'00.0"N 99°20'24.0"W |
| 272 | MN181714.1 | 45°27'00.0"N 99°20'24.0"W |
| 273 | MN181713.1 | 45°27'00.0"N 99°20'24.0"W |
| 274 | MN181712.1 | 30°58'48.0"N 84°31'48.0"W |
| 275 | MN181711.1 | 30°58'48.0"N 84°31'48.0"W |
| 276 | MN181706.1 | 30°58'48.0"N 84°31'48.0"W |
| 277 | MN181707.1 | 30°58'48.0"N 84°31'48.0"W |
| 278 | MN181708.1 | 30°58'48.0"N 84°31'48.0"W |
| 279 | MN181709.1 | 30°58'48.0"N 84°31'48.0"W |
| 280 | MN181710.1 | 30°58'48.0"N 84°31'48.0"W |
| 281 | MN181701.1 | 30°58'48.0"N 84°31'48.0"W |
| 282 | MN181702.1 | 30°58'48.0"N 84°31'48.0"W |
| 283 | MN181703.1 | 29°37'48.0"N 82°25'12.0"W |
| 284 | MN181704.1 | 29°37'48.0"N 82°25'12.0"W |
| 285 | MN181705.1 | 29°37'48.0"N 82°25'12.0"W |
| 286 | MN181700.1 | 29°37'48.0"N 82°25'12.0"W |
| 287 | MN181699.1 | 29°37'48.0"N 82°25'12.0"W |
| 288 | MN181698.1 | 29°37'48.0"N 82°25'12.0"W |
| 289 | MN181697.1 | 29°37'48.0"N 82°25'12.0"W |
| 290 | MN181696.1 | 29°37'48.0"N 82°25'12.0"W |
| 291 | MN181694.1 | 29°37'48.0"N 82°25'12.0"W |
| 292 | MN181695.1 | 29°37'48.0"N 82°25'12.0"W |
| 293 | MN181689.1 | 42°36'36.0"N 85°38'24.0"W |
| 294 | MN181686.1 | 42°36'36.0"N 85°38'24.0"W |
| 295 | MN181687.1 | 42°36'36.0"N 85°38'24.0"W |
| 296 | MN181688.1 | 42°36'36.0"N 85°38'24.0"W |
| 297 | MN181683.1 | 42°36'36.0"N 85°38'24.0"W |
| 298 | MN181682.1 | 42°36'36.0"N 85°38'24.0"W |
| 299 | MN181681.1 | 42°36'36.0"N 85°38'24.0"W |
| 300 | MN181676.1 | 42°36'36.0"N 85°38'24.0"W |

| Sl. No | Accession | GPS | Country |
| --- | --- | --- | --- |
| 301 | MN181677.1 | 47°21'00.0"N 118°01'12.0"W | USA |
| 302 | MN181678.1 | 47°21'00.0"N 118°01'12.0"W |
| 303 | MN181680.1 | 47°21'00.0"N 118°01'12.0"W |
| 304 | MN181679.1 | 47°21'00.0"N 118°01'12.0"W |
| 305 | MN181672.1 | 47°21'00.0"N 118°01'12.0"W |
| 306 | MN181674.1 | 47°21'00.0"N 118°01'12.0"W |
| 307 | MN181675.1 | 47°21'00.0"N 118°01'12.0"W |
| 308 | MN181670.1 | 47°21'00.0"N 118°01'12.0"W |
| 309 | MN181669.1 | 41°45'36.0"N 72°40'12.0"W |
| 310 | MN181668.1 | 41°45'36.0"N 72°40'12.0"W |
| 311 | MN181667.1 | 41°45'36.0"N 72°40'12.0"W |
| 312 | MN181666.1 | 41°45'36.0"N 72°40'12.0"W |
| 313 | MN181661.1 | 41°45'36.0"N 72°40'12.0"W |
| 314 | MN181662.1 | 41°45'36.0"N 72°40'12.0"W |
| 315 | MN181663.1 | 41°45'36.0"N 72°40'12.0"W |
| 316 | MN181664.1 | 41°45'36.0"N 72°40'12.0"W |
| 317 | MN181665.1 | 41°45'36.0"N 72°40'12.0"W |
| 318 | MN181656.1 | 41°45'36.0"N 72°40'12.0"W |
| 319 | MN181657.1 | 41°45'36.0"N 72°40'12.0"W |
| 320 | MN181658.1 | 42°36'36.0"N 85°38'24.0"W |
| 321 | MN181659.1 | 42°36'36.0"N 85°38'24.0"W |
| 322 | MN181660.1 | 42°36'36.0"N 85°38'24.0"W |
| 323 | MN181651.1 | 42°36'36.0"N 85°38'24.0"W |
| 324 | MN181652.1 | 42°36'36.0"N 85°38'24.0"W |
| 325 | MN181653.1 | 42°36'36.0"N 85°38'24.0"W |
| 326 | MN181654.1 | 42°36'36.0"N 85°38'24.0"W |
| 327 | MN181655.1 | 42°36'36.0"N 85°38'24.0"W |
| 328 | MN181646.1 | 42°36'36.0"N 85°38'24.0"W |
| 329 | MN181648.1 | 42°36'36.0"N 85°38'24.0"W |
| 330 | MN181649.1 | 42°36'36.0"N 85°38'24.0"W |
| 331 | MN181650.1 | 42°36'36.0"N 85°38'24.0"W |
| 332 | MN181644.1 | 39°19'12.0"N 77°16'48.0"W |
| 333 | MN181643.1 | 39°19'12.0"N 77°16'48.0"W |
| 334 | MN181642.1 | 39°19'12.0"N 77°16'48.0"W |
| 335 | MN181641.1 | 39°19'12.0"N 77°16'48.0"W |
| 336 | MN181636.1 | 39°19'12.0"N 77°16'48.0"W |
| 337 | MN181637.1 | 39°19'12.0"N 77°16'48.0"W |
| 338 | MN181638.1 | 39°19'12.0"N 77°16'48.0"W |
| 339 | MN181640.1 | 39°19'12.0"N 77°16'48.0"W |
| 340 | MN181639.1 | 39°19'12.0"N 77°16'48.0"W |
| 341 | MN181635.1 | 39°19'12.0"N 77°16'48.0"W |
| 342 | MN181631.1 | 39°19'12.0"N 77°16'48.0"W |
| 343 | MN181632.1 | 39°19'12.0"N 77°16'48.0"W |
| 344 | MN181634.1 | 38°17'24.0"N 85°37'48.0"W |
| 345 | MN181633.1 | 38°17'24.0"N 85°37'48.0"W |
| 346 | MN181626.1 | 38°17'24.0"N 85°37'48.0"W |
| 347 | MN181627.1 | 38°17'24.0"N 85°37'48.0"W |
| 348 | MN181628.1 | 38°17'24.0"N 85°37'48.0"W |
| 349 | MN181629.1 | 38°17'24.0"N 85°37'48.0"W |
| 350 | MN181630.1 | 38°17'24.0"N 85°37'48.0"W |
| 351 | MN181621.1 | 38°17'24.0"N 85°37'48.0"W |
| 352 | MN181622.1 | 38°17'24.0"N 85°37'48.0"W |
| 353 | MN181623.1 | 38°17'24.0"N 85°37'48.0"W |
| 354 | MN181624.1 | 38°17'24.0"N 85°37'48.0"W |
| 355 | MN181625.1 | 38°17'24.0"N 85°37'48.0"W |
| 356 | JF283398.1 | 38°17'24.0"N 85°37'48.0"W |

| Sl. No | Accession | GPS | Country |
| --- | --- | --- | --- |
| 1 | JN276270.1 | 35°15'39.6"S 149°03'32.4"E | Australia |
| 2 | MN182044.1 | 37°54'00.0"S 145°16'48.0"E |
| 3 | MN182043.1 | 37°54'00.0"S 145°16'48.0"E |
| 4 | MN182042.1 | 37°54'00.0"S 145°16'48.0"E |
| 5 | MN182039.1 | 37°54'00.0"S 145°16'48.0"E |
| 6 | MN182038.1 | 37°43'12.0"S 145°03'00.0"E |
| 7 | MN182020.1 | 37°43'12.0"S 145°03'00.0"E |
| 8 | MN182016.1 | 37°43'12.0"S 145°03'00.0"E |
| 9 | MN182014.1 | 37°43'12.0"S 145°03'00.0"E |
| 10 | MN182001.1 | 26°25'48.0"S 152°45'36.0"E |
| 11 | MN182000.1 | 26°25'48.0"S 152°45'36.0"E |
| 12 | MN181999.1 | 26°25'48.0"S 152°45'36.0"E |
| 13 | MN181998.1 | 26°25'48.0"S 152°45'36.0"E |
| 14 | MN181997.1 | 37°12'36.0"S 145°00'00.0"E |
| 15 | MN181993.1 | 37°12'36.0"S 145°00'00.0"E |
| 16 | MN181992.1 | 37°12'36.0"S 145°00'00.0"E |
| 17 | MN181991.1 | 37°12'36.0"S 145°00'00.0"E |
| 18 | MN181990.1 | 37°12'36.0"S 145°00'00.0"E |
| 19 | MN181989.1 | 37°12'36.0"S 145°00'00.0"E |
| 20 | MN181956.1 | 41°12'00.0"S 146°21'36.0"E |
| 21 | MN181955.1 | 41°12'00.0"S 146°21'36.0"E |
| 22 | MN181954.1 | 41°12'00.0"S 146°21'36.0"E |
| 23 | MN181953.1 | 41°12'00.0"S 146°21'36.0"E |
| 24 | MN181952.1 | 41°12'00.0"S 146°21'36.0"E |
| 25 | MN181951.1 | 41°12'00.0"S 146°21'36.0"E |
| 26 | MN181950.1 | 41°12'00.0"S 146°21'36.0"E |
| 27 | MN181949.1 | 41°12'00.0"S 146°21'36.0"E |
| 28 | MN181948.1 | 41°12'00.0"S 146°21'36.0"E |
| 29 | MN181947.1 | 41°12'00.0"S 146°21'36.0"E |
| 30 | MN181875.1 | 41°12'00.0"S 146°21'36.0"E |
| 31 | MN181874.1 | 41°12'00.0"S 146°21'36.0"E |
| 32 | MN181845.1 | 37°53'24.0"S 145°20'24.0"E |
| 33 | MN181841.1 | 37°53'24.0"S 145°20'24.0"E |
| 34 | MN181842.1 | 37°53'24.0"S 145°20'24.0"E |
| 35 | MN181844.1 | 37°53'24.0"S 145°20'24.0"E |
| 36 | MN181843.1 | 34°44'24.0"S 150°49'12.0"E |
| 37 | MN181840.1 | 34°44'24.0"S 150°49'12.0"E |
| 38 | MN181839.1 | 34°44'24.0"S 150°49'12.0"E |
| 39 | MN181838.1 | 34°44'24.0"S 150°49'12.0"E |
| 40 | MN181837.1 | 34°44'24.0"S 150°49'12.0"E |
| 41 | MN181836.1 | 34°44'24.0"S 150°49'12.0"E |
| 42 | MN181835.1 | 34°46'48.0"S 150°43'48.0"E |
| 43 | MN181834.1 | 34°46'48.0"S 150°43'48.0"E |
| 44 | MN181833.1 | 34°46'48.0"S 150°43'48.0"E |
| 45 | MN181823.1 | 34°46'48.0"S 150°43'48.0"E |
| 46 | MN181822.1 | 37°51'36.0"S 145°04'12.0"E |
| 47 | MN181821.1 | 37°51'36.0"S 145°04'12.0"E |
| 48 | MN181820.1 | 37°51'36.0"S 145°04'12.0"E |
| 49 | MN181819.1 | 37°51'36.0"S 145°04'12.0"E |
| 50 | MN181818.1 | 26°25'48.0"S 152°45'36.0"E |
| 51 | MN181817.1 | 26°25'48.0"S 152°45'36.0"E |
| 52 | MN181816.1 | 26°25'48.0"S 152°45'36.0"E |
| 53 | MN181815.1 | 26°25'48.0"S 152°45'36.0"E |
| 54 | MN181814.1 | 26°25'48.0"S 152°45'36.0"E |
| 55 | MN181813.1 | 26°25'48.0"S 152°45'36.0"E |
| 56 | MN181812.1 | 26°25'48.0"S 152°45'36.0"E |
| 57 | MN181811.1 | 26°25'48.0"S 152°45'36.0"E |
| 58 | MN181809.1 | 26°25'48.0"S 152°45'36.0"E |
| 59 | MN181808.1 | 26°25'48.0"S 152°45'36.0"E |
| 60 | MN181807.1 | 37°01'12.0"S 145°08'24.0"E |
| 61 | MN181806.1 | 37°01'12.0"S 145°08'24.0"E |
| 62 | MN181805.1 | 37°01'12.0"S 145°08'24.0"E |
| 63 | MN181804.1 | 37°01'12.0"S 145°08'24.0"E |
| 64 | MN181803.1 | 37°01'12.0"S 145°08'24.0"E |
| 65 | MN181802.1 | 35°15'36.0"S 149°03'36.0"E |
| 66 | MN181801.1 | 35°15'36.0"S 149°03'36.0"E |
| 67 | MN181800.1 | 35°15'36.0"S 149°03'36.0"E |
| 68 | MN181799.1 | 35°15'36.0"S 149°03'36.0"E |
| 69 | MN181798.1 | 35°15'36.0"S 149°03'36.0"E |
| 70 | MN181797.1 | 31°59'24.0"S 115°54'00.0"E |
| 71 | MN181796.1 | 31°59'24.0"S 115°54'00.0"E |
| 72 | MN181795.1 | 31°59'24.0"S 115°54'00.0"E |
| 73 | MN181794.1 | 31°59'24.0"S 115°54'00.0"E |
| 74 | MN181793.1 | 31°59'24.0"S 115°54'00.0"E |
| 75 | KF404991.1 | 38°03'00.0"S 145°41'02.4"E |
| 76 | KF396514.1 | 38°03'00.0"S 145°41'02.4"E |
| 77 | MN182050.1 | 37°09'00.0"S 175°33'36.0"E | New Zealand |
| 78 | MN182049.1 | 37°09'00.0"S 175°33'36.0"E |
| 79 | MN182048.1 | 37°09'00.0"S 175°33'36.0"E |
| 80 | MN181988.1 | 37°09'00.0"S 175°33'36.0"E |
| 81 | MN181987.1 | 37°09'00.0"S 175°33'36.0"E |
| 82 | MN182019.1 | 37°25'12.0"S 175°55'48.0"E |
| 83 | MN181964.1 | 43°31'12.0"S 172°34'48.0"E |
| 84 | MN181957.1 | 43°31'12.0"S 172°34'48.0"E |
| 85 | MN181958.1 | 43°31'12.0"S 172°34'48.0"E |
| 86 | MN181960.1 | 43°31'12.0"S 172°34'48.0"E |
| 87 | MN181959.1 | 43°31'12.0"S 172°34'48.0"E |
| 88 | MN181961.1 | 43°31'12.0"S 172°34'48.0"E |
| 89 | MN181962.1 | 43°31'12.0"S 172°34'48.0"E |
| 90 | MN181963.1 | 43°31'12.0"S 172°34'48.0"E |
| 91 | MN181846.1 | 43°31'12.0"S 172°34'48.0"E |
| 92 | MN181832.1 | 43°31'12.0"S 172°34'48.0"E |
| 93 | MN181831.1 | 43°31'12.0"S 172°34'48.0"E |
| 94 | MN181830.1 | 43°31'12.0"S 172°34'48.0"E |
| 95 | MN181829.1 | 43°31'12.0"S 172°34'48.0"E |
| 96 | MN181828.1 | 43°31'12.0"S 172°34'48.0"E |
| 97 | MN181827.1 | 43°31'12.0"S 172°34'48.0"E |
| 98 | MN181826.1 | 43°31'12.0"S 172°34'48.0"E |
| 99 | MN181825.1 | 43°31'12.0"S 172°34'48.0"E |
| 100 | MN181824.1 | 43°31'12.0"S 172°34'48.0"E |
| 101 | MN181810.1 | 43°31'48.0"S 172°33'36.0"E |
