# Supplementary material for: Phylogeography and genetic diversity of Pieris rapae across continents
Source: PLoS One. 2026 Jul 24;21(7):e0354257. doi: 10.1371/journal.pone.0354257 (PMC13399322; doi:10.1371/journal.pone.0354257)
Supplement: Attachment — Submitted filename: Response to Reviewers.docx [file pone.0354257.s003.docx]

03/07/2026

To

Academic Editor

PLOS One

Sub: Response to the Reviewers and Editor’s comments for the Reference PONE-D-26-28617R1.

We sincerely thank the editor and reviewer for their careful reading and valuable suggestions regarding our manuscript “Phylogeography and Genetic Diversity of Pieris rapae Across Continents”. We have provided all responses to Editor and Reviewer.

**Response to Editor**

We sincerely thank the Academic Editor for the careful evaluation of our manuscript and for the constructive comments. We have carefully addressed all editorial requirements as follows:

**Comment 1:** Please ensure that your manuscript meets PLOS ONE's style requirements, including those for file naming.

**Response:** We have carefully revised the manuscript to comply with the PLOS ONE formatting guidelines and file naming requirements. The manuscript has been prepared according to the journal's style templates.

**Comment 2:** Please provide a complete Data Availability Statement in the submission form.

**Response:** We have provided a complete Data Availability Statement in the submission system. The statement has been updated to accurately reflect the availability of the data used in this study.

**Comment 3:** Please upload a new copy of Figures 1 and 2 as the detail is not clear.

**Response:** We have prepared and uploaded new high-resolution versions of Figures 1 and 2 to improve image clarity and readability in accordance with the PLOS ONE figure guidelines.

**Comment 4:** If the reviewer comments include a recommendation to cite specific previously published works, please review and evaluate these publications to determine whether they are relevant and should be cited.

**Response:** We carefully reviewed the reviewer’s recommendations and the relevant literature. Appropriate comparisons with previous studies have been incorporated into the Discussion where relevant.

**Comment 5:** Please review your reference list to ensure that it is complete and correct. If you have cited papers that have been retracted, please include the rationale for doing so or remove them.

**Response:** We have carefully reviewed the reference list to ensure that all citations are complete, accurate, and up to date. We confirm that no retracted articles are cited in the revised manuscript.

**Response to Reviewer**

**Reviewer Comment 1:** The Methods section should provide additional details regarding how sequence redundancy was handled and how sequence quality control was performed. This information is important for assessing the reliability and reproducibility of the study.

**My Response:** We thank the reviewer for this valuable suggestion. In response, we have revised the **Sequence retrieval and sample selection and Genetic diversity and phylogeographic analyses** sections of the Materials and Methods to provide a more detailed description of our sequence screening and quality control procedures. Specifically, we clarified the criteria used for sequence inclusion, including sequence length, sequence quality, ambiguous nucleotide filtering, removal of duplicate or redundant records, and exclusion of sequences with incomplete metadata or uncertain species identification. We also expanded the description of the alignment quality control process by specifying the manual inspection and trimming of poor-quality regions and uneven sequence termini in BioEdit, followed by validation of the curated alignment in MEGA prior to downstream analyses. These revisions improve the transparency, reproducibility, and methodological clarity of the study.

**Reviewer Comment 2:** The authors should discuss the potential effects of unequal sampling among continents. Differences in sample size may influence estimates of haplotype diversity and the interpretation of phylogeographic patterns.

**My Response:** We sincerely thank the reviewer for this constructive suggestion. We have revised the Discussion to acknowledge the potential influence of unequal sampling effort among continents on estimates of genetic diversity and the interpretation of phylogeographic patterns. Specifically, we now discuss that the larger numbers of COI sequences from Europe and North America may have increased the likelihood of detecting additional haplotypes, whereas the comparatively smaller sample sizes from Africa and Oceania may have underestimated regional genetic diversity. We further emphasize that differences in haplotype richness should therefore be interpreted with caution and note that future studies incorporating more balanced geographic sampling will provide a more comprehensive understanding of the global genetic diversity and phylogeographic structure of Pieris rapae. These revisions strengthen the interpretation of our findings by explicitly acknowledging the potential effects of sampling bias.

**Reviewer Comment 3:** The description of the network analysis should be clarified. The authors should clearly indicate which network method was used and ensure that the terminology is consistent throughout the Methods, Results, and figure legends.

**My Response:** We thank the reviewer for identifying this inconsistency. We have carefully revised the manuscript to ensure consistent terminology describing the haplotype network analysis. Specifically, we clarified that a **minimum spanning network (MSN)** was constructed using PopART v1.7 and have replaced the inconsistent terminology throughout the **Materials and Methods, Results,** and **Figure 1 legend**. These revisions provide a clear and consistent description of the network analysis performed in this study.

**Reviewer Comment 4:** Some conclusions should be presented more cautiously. Inferences regarding introduction history and source populations should be interpreted in light of the fact that the analyses are based solely on mitochondrial COI data.

**My Response:** We sincerely thank the reviewer for this important and constructive comment. In response, we have revised the Discussion to present our conclusions more cautiously and to explicitly acknowledge the limitations of relying on a single mitochondrial marker. Specifically, we have modified statements concerning the introduction history, source populations, and interpretation of major haplotypes to avoid overstatement and to emphasize that these inferences are based solely on mitochondrial COI data. We have also revised the concluding paragraph to state that the inferred invasion history and source populations should be interpreted with caution and added a recommendation that future studies integrating nuclear genomic markers with more balanced geographic sampling will provide a more comprehensive understanding of the global dispersal history, admixture, and population structure of Pieris rapae. These revisions ensure that our conclusions are fully supported by the available evidence while acknowledging the inherent limitations of mitochondrial data.

**Reviewer Comment 5:** The Discussion could be strengthened by comparing the present findings with previous studies on the global invasion history of *Pieris rapae.* In addition, careful proofreading and verification of tables and figure captions would improve the overall presentation of the manuscript.

**My Response:** We sincerely thank the reviewer for this helpful suggestion. We have revised the Discussion to strengthen the comparison of our findings with previous studies on the global invasion history of Pieris rapae. Specifically, we added a comparison highlighting that the high genetic diversity observed in Europe is consistent with previous studies identifying the Mediterranean–West Asian region as the native range of P. rapae and proposing Europe as an important contributor to its subsequent global colonization through natural and human-mediated dispersal. We also compared our observed phylogeographic patterns with earlier evidence supporting the role of long-distance, human-mediated dispersal in the worldwide expansion of the species. In addition, we carefully proofread the entire manuscript and verified all tables and figure captions to improve clarity, consistency, and overall presentation. All identified typographical, grammatical, and formatting issues have been corrected.
