# Supplementary material for: Phylogeography and genetic diversity of Pieris rapae across continents
Source: PLoS One. 2026 Jul 24;21(7):e0354257. doi: 10.1371/journal.pone.0354257 (PMC13399322; doi:10.1371/journal.pone.0354257)
Supplement: Peer review history

# Peer review: Decision Letter 0

**DOI:** 10.1371/journal.pone.0354257.r001

**Authors:** Taslima Sheikh

30 Jun 2026

PONE-D-26-28617

Phylogeography and Genetic Diversity of Pieris rapae Across Continents

PLOS One

Dear Dr. Siam,

Thank you for submitting your manuscript to PLOS ONE. After careful consideration, we feel that it has merit but does not fully meet PLOS ONE’s publication criteria as it currently stands. Therefore, we invite you to submit a revised version of the manuscript that addresses the points raised during the review process.

Please submit your revised manuscript by Jul 30 2026 11:59PM. If you will need more time than this to complete your revisions, please reply to this message or contact the journal office at plosone@plos.org. When you're ready to submit your revision, log on to https://www.editorialmanager.com/pone/ and select the 'Submissions Needing Revision' folder to locate your manuscript file.

Please include the following items when submitting your revised manuscript:

- A letter that responds to each point raised by the academic editor and reviewer(s). You should upload this letter as a separate file labeled 'Response to Reviewers'.
- A marked-up copy of your manuscript that highlights changes made to the original version. You should upload this as a separate file labeled 'Revised Manuscript with Track Changes'.
- An unmarked version of your revised paper without tracked changes. You should upload this as a separate file labeled 'Manuscript'.

If you would like to make changes to your financial disclosure, please include your updated statement in your cover letter. Guidelines for resubmitting your figure files are available below the reviewer comments at the end of this letter.

If applicable, we recommend that you deposit your laboratory protocols in protocols.io to enhance the reproducibility of your results. Protocols.io assigns your protocol its own identifier (DOI) so that it can be cited independently in the future. For instructions see: https://journals.plos.org/plosone/s/submission-guidelines#loc-laboratory-protocols. Additionally, PLOS ONE offers an option for publishing peer-reviewed Lab Protocol articles, which describe protocols hosted on protocols.io. Read more information on sharing protocols at https://plos.org/protocols?utm_medium=editorial-email&utm_source=authorletters&utm_campaign=protocols.

As the corresponding author, your ORCID iD is verified in the submission system and will appear in the published article. PLOS supports the use of ORCID, and we encourage all coauthors to register for an ORCID iD and use it as well. Please encourage your coauthors to verify their ORCID iD within the submission system before final acceptance, as unverified ORCID iDs will not appear in the published article. *Only*  the individual author can complete the verification step; PLOS staff *cannot*  verify ORCID iDs on behalf of authors.

We look forward to receiving your revised manuscript.

Kind regards,

Taslima Sheikh

Academic Editor

PLOS One

Journal Requirements:

When submitting your revision, we need you to address these additional requirements.

1. Please ensure that your manuscript meets PLOS ONE's style requirements, including those for file naming. The PLOS ONE style templates can be found at

https://journals.plos.org/plosone/s/file?id=wjVg/PLOSOne_formatting_sample_main_body.pdf and

https://journals.plos.org/plosone/s/file?id=ba62/PLOSOne_formatting_sample_title_authors_affiliations.pdf

2. Please provide a complete Data Availability Statement in the submission form, ensuring you include all necessary access information or a reason for why you are unable to make your data freely accessible. If your research concerns only data provided within your submission, please write "All data are in the manuscript and/or supporting information files" as your Data Availability Statement.

3. Please upload a new copy of Figures 1 and 2 as the detail is not clear. Please follow the link for more information:  https://journals.plos.org/plosone/s/figures

4. If the reviewer comments include a recommendation to cite specific previously published works, please review and evaluate these publications to determine whether they are relevant and should be cited. There is no requirement to cite these works unless the editor has indicated otherwise.

5. Please review your reference list to ensure that it is complete and correct. If you have cited papers that have been retracted, please include the rationale for doing so in the manuscript text, or remove these references and replace them with relevant current references. Any changes to the reference list should be mentioned in the rebuttal letter that accompanies your revised manuscript. If you need to cite a retracted article, indicate the article’s retracted status in the References list and also include a citation and full reference for the retraction notice.

[Note: HTML markup is below. Please do not edit.]

Reviewers' comments:

Reviewer's Responses to Questions

**Comments to the Author**

1. Is the manuscript technically sound, and do the data support the conclusions?

The manuscript must describe a technically sound piece of scientific research with data that supports the conclusions. Experiments must have been conducted rigorously, with appropriate controls, replication, and sample sizes. The conclusions must be drawn appropriately based on the data presented.

Reviewer #1: Yes

**********

2. Has the statistical analysis been performed appropriately and rigorously?

Reviewer #1: Yes

**********

3. Have the authors made all data underlying the findings in their manuscript fully available?

The PLOS Data policy requires authors to make all data underlying the findings described in their manuscript fully available without restriction, with rare exception (please refer to the Data Availability Statement in the manuscript PDF file). The data should be provided as part of the manuscript or its supporting information, or deposited to a public repository. For example, in addition to summary statistics, the data points behind means, medians and variance measures should be available. If there are restrictions on publicly sharing data—e.g. participant privacy or use of data from a third party—those must be specified.

Reviewer #1: Yes

**********

4. Is the manuscript presented in an intelligible fashion and written in standard English?

PLOS ONE does not copyedit accepted manuscripts, so the language in submitted articles must be clear, correct, and unambiguous. Any typographical or grammatical errors should be corrected at revision, so please note any specific errors here.

Reviewer #1: Yes

**********

5. Review Comments to the Author

Please use the space provided to explain your answers to the questions above. You may also include additional comments for the author, including concerns about dual publication, research ethics, or publication ethics. (Please upload your review as an attachment if it exceeds 20,000 characters)

Reviewer #1: Overall, this manuscript provides a valuable global analysis of the genetic diversity and phylogeography of Pieris rapae, and I recommend its acceptance after revision. The following points should be addressed to further improve the quality and clarity of the study:

1. The Methods section should provide additional details regarding how sequence redundancy was handled and how sequence quality control was performed. This information is important for assessing the reliability and reproducibility of the study.

2. The authors should discuss the potential effects of unequal sampling among continents. Differences in sample size may influence estimates of haplotype diversity and the interpretation of phylogeographic patterns.

3. The description of the network analysis should be clarified. The authors should clearly indicate which network method was used and ensure that the terminology is consistent throughout the Methods, Results, and figure legends.

4. Some conclusions should be presented more cautiously. Inferences regarding introduction history and source populations should be interpreted in light of the fact that the analyses are based solely on mitochondrial COI data.

5. The Discussion could be strengthened by comparing the present findings with previous studies on the global invasion history of Pieris rapae. In addition, careful proofreading and verification of tables and figure captions would improve the overall presentation of the manuscript.

**********

6. PLOS authors have the option to publish the peer review history of their article (what does this mean?). If published, this will include your full peer review and any attached files.

If you choose “no”, your identity will remain anonymous but your review may still be made public.

**Do you want your identity to be public for this peer review?**  For information about this choice, including consent withdrawal, please see our Privacy Policy.

Reviewer #1: **Yes:**  LOVISH GARLANI

**********

[NOTE: If reviewer comments were submitted as an attachment file, they will be attached to this email and accessible via the submission site. Please log into your account, locate the manuscript record, and check for the action link "View Attachments". If this link does not appear, there are no attachment files.]

To ensure your figures meet our technical requirements, please review our figure guidelines: https://journals.plos.org/plosone/s/figures

You may also use PLOS’s free figure tool, NAAS, to help you prepare publication quality figures: https://journals.plos.org/plosone/s/figures#loc-tools-for-figure-preparation.

NAAS will assess whether your figures meet our technical requirements by comparing each figure against our figure specifications.

# Author comment: Author response to Decision Letter 1

**DOI:** 10.1371/journal.pone.0354257.r002

3 Jul 2026

27/06/2026

To

Academic Editor

PLOS One

Sub: Response to the Reviewers and Editor’s comments for the Reference PONE-D-26-28617.

We sincerely thank the editor and reviewer for their careful reading and valuable suggestions regarding our manuscript “Phylogeography and Genetic Diversity of Pieris rapae Across Continents”. We have provided all responses to Editor and Reviewer.

Response to Editor

We sincerely thank the Academic Editor for the careful evaluation of our manuscript and for the constructive comments. We have carefully addressed all editorial requirements as follows:

Comment 1: Please ensure that your manuscript meets PLOS ONE's style requirements, including those for file naming.

My Response: We have carefully revised the manuscript to comply with the PLOS ONE formatting guidelines and file naming requirements. The manuscript has been prepared according to the journal's style templates.

Comment 2: Please provide a complete Data Availability Statement in the submission form.

My Response: We have provided a complete Data Availability Statement in the submission system. The statement has been updated to accurately reflect the availability of the data used in this study.

Comment 3: Please upload a new copy of Figures 1 and 2 as the detail is not clear.

My Response: We have prepared and uploaded new high-resolution versions of Figures 1 and 2 to improve image clarity and readability in accordance with the PLOS ONE figure guidelines.

Comment 4: If the reviewer comments include a recommendation to cite specific previously published works, please review and evaluate these publications to determine whether they are relevant and should be cited.

My Response: We carefully reviewed the reviewer’s recommendations and the relevant literature. Appropriate comparisons with previous studies have been incorporated into the Discussion where relevant.

Comment 5: Please review your reference list to ensure that it is complete and correct. If you have cited papers that have been retracted, please include the rationale for doing so or remove them.

My Response: We have carefully reviewed the reference list to ensure that all citations are complete, accurate, and up to date. We confirm that no retracted articles are cited in the revised manuscript.

Response to Reviewer

Reviewer Comment 1: The Methods section should provide additional details regarding how sequence redundancy was handled and how sequence quality control was performed. This information is important for assessing the reliability and reproducibility of the study.

My Response: We thank the reviewer for this valuable suggestion. In response, we have revised the Sequence retrieval and sample selection and Genetic diversity and phylogeographic analyses sections of the Materials and Methods to provide a more detailed description of our sequence screening and quality control procedures. Specifically, we clarified the criteria used for sequence inclusion, including sequence length, sequence quality, ambiguous nucleotide filtering, removal of duplicate or redundant records, and exclusion of sequences with incomplete metadata or uncertain species identification. We also expanded the description of the alignment quality control process by specifying the manual inspection and trimming of poor-quality regions and uneven sequence termini in BioEdit, followed by validation of the curated alignment in MEGA prior to downstream analyses. These revisions improve the transparency, reproducibility, and methodological clarity of the study.

Reviewer Comment 2: The authors should discuss the potential effects of unequal sampling among continents. Differences in sample size may influence estimates of haplotype diversity and the interpretation of phylogeographic patterns.

My Response: We sincerely thank the reviewer for this constructive suggestion. We have revised the Discussion to acknowledge the potential influence of unequal sampling effort among continents on estimates of genetic diversity and the interpretation of phylogeographic patterns. Specifically, we now discuss that the larger numbers of COI sequences from Europe and North America may have increased the likelihood of detecting additional haplotypes, whereas the comparatively smaller sample sizes from Africa and Oceania may have underestimated regional genetic diversity. We further emphasize that differences in haplotype richness should therefore be interpreted with caution and note that future studies incorporating more balanced geographic sampling will provide a more comprehensive understanding of the global genetic diversity and phylogeographic structure of Pieris rapae. These revisions strengthen the interpretation of our findings by explicitly acknowledging the potential effects of sampling bias.

Reviewer Comment 3: The description of the network analysis should be clarified. The authors should clearly indicate which network method was used and ensure that the terminology is consistent throughout the Methods, Results, and figure legends.

My Response: We thank the reviewer for identifying this inconsistency. We have carefully revised the manuscript to ensure consistent terminology describing the haplotype network analysis. Specifically, we clarified that a minimum spanning network (MSN) was constructed using PopART v1.7 and have replaced the inconsistent terminology throughout the Materials and Methods, Results, and Figure 1 legend. These revisions provide a clear and consistent description of the network analysis performed in this study.

Reviewer Comment 4: Some conclusions should be presented more cautiously. Inferences regarding introduction history and source populations should be interpreted in light of the fact that the analyses are based solely on mitochondrial COI data.

My Response: We sincerely thank the reviewer for this important and constructive comment. In response, we have revised the Discussion to present our conclusions more cautiously and to explicitly acknowledge the limitations of relying on a single mitochondrial marker. Specifically, we have modified statements concerning the introduction history, source populations, and interpretation of major haplotypes to avoid overstatement and to emphasize that these inferences are based solely on mitochondrial COI data. We have also revised the concluding paragraph to state that the inferred invasion history and source populations should be interpreted with caution and added a recommendation that future studies integrating nuclear genomic markers with more balanced geographic sampling will provide a more comprehensive understanding of the global dispersal history, admixture, and population structure of Pieris rapae. These revisions ensure that our conclusions are fully supported by the available evidence while acknowledging the inherent limitations of mitochondrial data.

Reviewer Comment 5: The Discussion could be strengthened by comparing the present findings with previous studies on the global invasion history of Pieris rapae. In addition, careful proofreading and verification of tables and figure captions would improve the overall presentation of the manuscript.

My Response: We sincerely thank the reviewer for this helpful suggestion. We have revised the Discussion to strengthen the comparison of our findings with previous studies on the global invasion history of Pieris rapae. Specifically, we added a comparison highlighting that the high genetic diversity observed in Europe is consistent with previous studies identifying the Mediterranean–West Asian region as the native range of P. rapae and proposing Europe as an important contributor to its subsequent global colonization through natural and human-mediated dispersal. We also compared our observed phylogeographic patterns with earlier evidence supporting the role of long-distance, human-mediated dispersal in the worldwide expansion of the species. In addition, we carefully proofread the entire manuscript and verified all tables and figure captions to improve clarity, consistency, and overall presentation. All identified typographical, grammatical, and formatting issues have been corrected.

Attachment Submitted filename: Response to Reviewers.docx

# Editor report: Decision Letter 1

**DOI:** 10.1371/journal.pone.0354257.r003

**Authors:** Taslima Sheikh

6 Jul 2026

Phylogeography and Genetic Diversity of Pieris rapae Across Continents

PONE-D-26-28617R1

Dear Dr. Siam

We’re pleased to inform you that your manuscript has been judged scientifically suitable for publication and will be formally accepted for publication once it meets all outstanding technical requirements.

Within one week, you’ll receive an e-mail detailing the required amendments. When these have been addressed, you’ll receive a formal acceptance letter and your manuscript will be scheduled for publication.

An invoice will be generated when your article is formally accepted. Please note, if your institution has a publishing partnership with PLOS and your article meets the relevant criteria, all or part of your publication costs will be covered. Please make sure your user information is up-to-date by logging into Editorial Manager at Editorial Manager® and clicking the ‘Update My Information' link at the top of the page. For questions related to billing, please contact billing support.

If your institution or institutions have a press office, please notify them about your upcoming paper to help maximize its impact. If they’ll be preparing press materials, please inform our press team as soon as possible -- no later than 48 hours after receiving the formal acceptance. Your manuscript will remain under strict press embargo until 2 pm Eastern Time on the date of publication. For more information, please contact onepress@plos.org.

Kind regards,

Taslima Sheikh

Academic Editor

PLOS One

# Editor report: Acceptance letter

**DOI:** 10.1371/journal.pone.0354257.r004

**Authors:** Taslima Sheikh

PONE-D-26-28617R1

PLOS One

Dear Dr. Siam,

I'm pleased to inform you that your manuscript has been deemed suitable for publication in PLOS One. Congratulations! Your manuscript is now being handed over to our production team.

At this stage, our production department will prepare your paper for publication. This includes ensuring the following:

* All references, tables, and figures are properly cited

* All relevant supporting information is included in the manuscript submission,

* There are no issues that prevent the paper from being properly typeset

You will receive further instructions from the production team, including instructions on how to review your proof when it is ready. Please keep in mind that we are working through a large volume of accepted articles, so please give us a few days to review your paper and let you know the next and final steps.

Lastly, if your institution or institutions have a press office, please let them know about your upcoming paper now to help maximize its impact. If they'll be preparing press materials, please inform our press team within the next 48 hours. Your manuscript will remain under strict press embargo until 2 pm Eastern Time on the date of publication. For more information, please contact onepress@plos.org.

You will receive an invoice from PLOS for your publication fee after your manuscript has reached the completed accept phase. If you receive an email requesting payment before acceptance or for any other service, this may be a phishing scheme. Learn how to identify phishing emails and protect your accounts at https://explore.plos.org/phishing.

If we can help with anything else, please email us at customercare@plos.org.

Thank you for submitting your work to PLOS ONE and supporting open access.

Kind regards,

PLOS ONE Editorial Office Staff

on behalf of

Dr. Taslima Sheikh

Academic Editor

PLOS One
